# Molecular basis for inhibition of α-thrombin activity by bacterial lipopolysaccharides

**DOI:** 10.1038/s41598-026-46276-5

**Published:** 2026-03-27

**Authors:** André L. Lira, Katelyn C. Drew, Rodrigo L. M. Dantas, Jiaqing Pang, Owen J. T. McCarty

**Affiliations:** 1 Department of Biomedical Engineering, Oregon Health & Science University, Portland, 97239 USA; 2 Department of Science and Technology, Federal University of São Paulo, São José dos Campos, São Paulo, 12245-000 Brazil

**Keywords:** Lipopolysaccharide, Thrombin, Sepsis, Bacteria, Infection, Coagulation, Biochemistry, Microbiology

## Abstract

Dysregulation of the coagulation pathway is a hallmark of endotoxemia and Gram-negative sepsis, leading to disseminated intravascular coagulation resulting from aberrant thrombin generation. Bacterial components such as lipopolysaccharides (LPS) have been shown to indirectly contribute to thrombin generation by activating or assembling members of the coagulation cascade in an LPS-chemotype dependent manner. Conversely, studies have shown that LPS may directly inhibit thrombin activity through an undefined mechanism. We studied whether the LPS chemotypes from bacteria including *Escherichia coli* (O111:B4, O26:B6), *Klebsiella pneumoniae*, and *Pseudomonas aeruginosa* bind and regulate thrombin activity. We found that these LPS chemotypes bound thrombin exosites via their polysaccharide regions. Only the *E.coli* O26:B6 and *K.pneumoniae* LPS chemotypes induced global structural changes in thrombin. Both the monomeric forms of *E.coli* O26:B6 and *K.pneumoniae* LPS chemotypes reduced the catalytic efficiency of thrombin and thrombin-dependent fibrin polymerization in purified systems. The *E.coli* O26:B6 LPS chemotype retained anticoagulant activity in plasma as an Ca^2+^-stabilized aggregate form. Our data suggests that select LPS chemotypes bind and inhibit the proteolytic activity of thrombin in a manner dependent on their supramolecular state. The heterogeneity of physicochemical properties of bacterial envelope components may contribute to the dysregulation of the coagulation pathway during endotoxemia.

**Supplementary Information:**

The online version contains supplementary material available at 10.1038/s41598-026-46276-5.

## Introduction

The hallmarks of bacterial infection include the presence of pathogen-associated molecular patterns (PAMP) such as lipopolysaccharide (LPS)^1^. Recognized by toll-like receptors on innate immune cells, LPS is a key PAMP for triggering an effective immune response to Gram-negative bacteria^1,2^. Yet, the ability of LPS to regulate the activation or activity of members of the coagulation cascade coupled with excessive inflammation can lead to septic shock and organ failure^3^. It remains unclear whether this mechanistic shift from helpful to harmful is dominated by the concentration or type of LPS released into the bloodstream, which creates a challenge for the management of Gram-negative bacterial infections.

The biological activity of LPS is closely related to its chemical heterogeneity and supramolecular organization^4–6^. LPS is a glycolipid found in the outer leaflet of the outer membrane of Gram-negative bacteria and is a major structural component of the outer membrane^7^. Structurally, LPS exhibits a three-component molecular structure consisting of lipid A, which anchors the molecule in the bacterial membrane; the core oligosaccharide, which links lipid A to the O-antigen; and the O-antigen polysaccharide, which is highly variable and is partly responsible for producing different chemotypes that influence host–pathogen crosstalk^5^. Based on the presence and length of the O antigen, LPS is classified into three main chemotype groups: smooth LPS (S-LPS), which contains the full-length O antigen polysaccharide; semi-rough LPS (SR-LPS), characterized by truncated O antigen chains; and rough LPS (R-LPS), which lacks the O antigen entirely, exposing only the lipid A and core oligosaccharide regions^7,8^. These structural variations affect not only the molecular recognition by the host immune system but also the physicochemical properties of LPS, including its ability to self-organize into monomeric/oligomeric (premicellar), micellar, or bilayer-like supramolecular forms^3,5,6^. The self-assembly behavior of LPS is influenced by environmental factors such as ionic strength, pH, temperature, and the presence of divalent cations like Ca^2+^ and Mg^2+^^9,10^. In stable micellar aggregates, protein interactions are primarily limited to the carbohydrate portions of LPS, whereas monomeric forms expose both carbohydrate and lipid A domains for protein binding. While LPS is known to trigger coagulation indirectly via cellular pathways, its potential as a direct physical modulator of key coagulation enzymes remains largely unexplored. Yet, we still lack a comprehensive mechanistic understanding of how LPS from different bacterial species or strains affect the host response to infection including initiation of cytokine storm or activation of the blood coagulation cascade.

Thrombin is the primary effector of the coagulation cascade, a multifunctional serine protease that converts fibrinogen to fibrin, activates platelets, and modulates endothelial function^11^. Thrombin plays an essential role in hemostasis as well as inflammation and the complement system, thereby linking the coagulation cascade with innate immunity^11,12^. Thrombin is also an integral component of feedback loops that enhance and regulate parallel coagulation pathways. Thrombin is a serine protease made up of two highly positively charged surface regions, termed exosites 1 and 2 that occur at opposite ends of the enzyme structure^13^. Exosite 1 (ES1) interacts with substrates and cofactors such as fibrinogen and thrombomodulin. Exosite 2 (ES2) binds heparin and the platelet receptor GPIbα^14^.While both ES1 and ES2 are distinct structural features of thrombin, they are also share common electrostatic properties and overlapping interactions with substrates and endogenous regulatory ligands^14^. Given the polyanionic nature of LPS molecules, these cationic exosites represent potential binding sites for a direct LPS–thrombin interaction, which could sterically or electrostatically modulate substrate recognition. Thrombin activity and generation remain central to therapeutic strategies for treatment or prevention of cardiovascular diseases^15^. Yet, despite the known clinical link between endotoxemia and coagulopathy, it remains unknown whether specific bacterial pathogens and their chemically diverse LPS components directly affect thrombin activity and generation through such physical associations.

Here we study the effect of LPS from *Escherichia coli* (O111:B4, O26:B6), *Klebsiella pneumoniae*, and *Pseudomonas aeruginosa* on thrombin activity. We demonstrate that LPS modulates thrombin structure and activity in a chemotype dependent manner, providing an example of the effect of structural diversity of bacteria and the host response. We define the biophysical features by which circulating LPS alters thrombin activity and highlight that LPS thrombin interactions represent a previously unnoticed regulatory axis in coagulation, with implications for thromboinflammation during bacterial infections.

## Results

### LPS chemotypes form distinct supramolecular aggregates

**Fig. 1:**
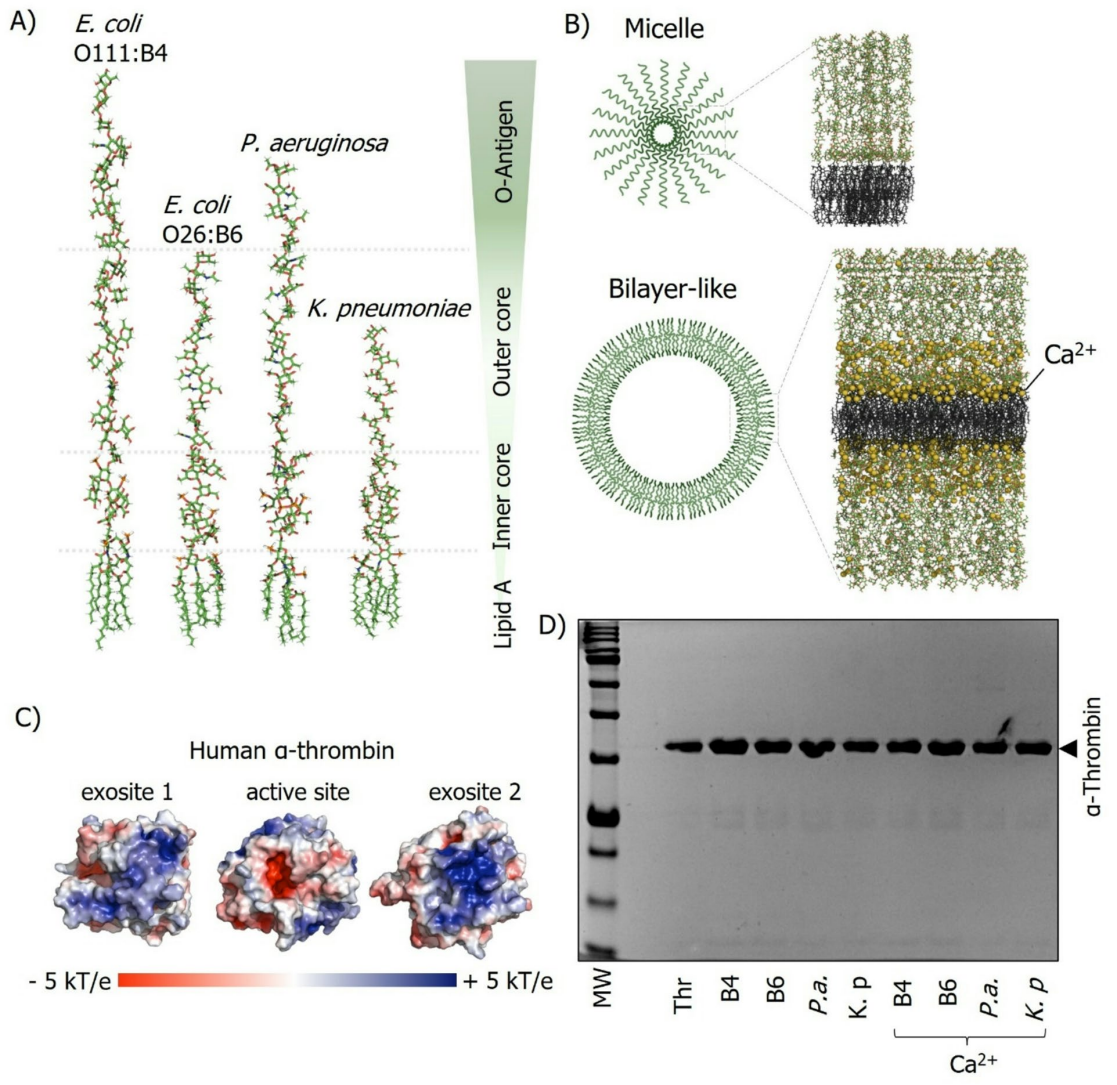
Structural modeling and biochemical characterization of LPS and human α-thrombin. (A) Molecular models of LPS from *E. coli* O111:B4 (S-LPS) and O26:B6 (SR-LPS), *P. aeruginosa* (S-LPS), and *K. pneumoniae* (SR-LPS), built using the CHARMM-GUI platform. (B) Schematic representations of LPS supramolecular assemblies (micelle and bilayer-like aggregate) generated using CHARMM-GUI and shown for conceptual illustration only. Ca^2+^ ions (yellow spheres) are included as an illustrative ion-bridging/charge-screening motif in the bilayer-like schematic and are not intended to imply experimentally or computationally validated aggregate stability. (C) Electrostatic surface potential of human α-thrombin (PDB: 1PPB). (D) SDS-PAGE analysis showing that thrombin is not degraded upon LPS exposure. Thrombin samples in HEPES buffer were incubated with LPS (50 µg/mL), prepared either with or without Ca^2+^, from the indicated Gram-negative bacteria for 15 min at 37 °C, followed by the addition of gel sample buffer and separation on 10% polyacrylamide gels. Lanes: MW, molecular weight marker; Thr, thrombin alone; B4, Thr+O111:B4; B6, Thr+O26:B6; *P.a*., Thr + *P. aeruginosa; K.p.*, Thr + *K. pneumoniae*, alone or in the presence of Ca^2+^.

We characterized the supramolecular organization of the selected LPS chemotypes by measuring critical micelle concentration (CMC), hydrodynamic diameter (DLS), and ζ-potential (Table 1; **Fig. ****S1****–S2**). The chemical structures of the LPS chemotypes analyzed, as well as schematic illustrations of representative micellar and bilayer-like supramolecular aggregates, are shown in Fig. 1A–B. CMC values determined by NPN fluorescence were 24 ± 1 µg/mL for *E. coli* O111:B4, 18 ± 2 µg/mL for *E. coli* O26:B6, 23 ± 4 µg/mL for *P. aeruginosa*, and 17 ± 7 µg/mL for *K. pneumoniae* (Table 1). The pre-assembly of LPS with CaCl_2_ caused aggregation and cross-linking, making it impossible to reliably determine the CMC under Ca^2+^-stabilized conditions^4^. DLS measurements revealed chemotype-dependent aggregate size distributions (Fig. S2), with apparent hydrodynamic diameters (d_h_) of 82 ± 5 nm (O111:B4), 223 ± 14 nm (O26:B6), 213 ± 31 nm (*P. aeruginosa*), and 92 ± 11 nm (*K. pneumoniae*) in buffer (Table 1). The presence of Ca^2+^ increased aggregate size for all chemotypes to 129 ± 9, 342 ± 34, 339 ± 44, and 163 ± 14 nm, respectively (Table 1). ζ-potentials in buffer were − 13 ± 3, − 27 ± 4, − 35 ± 5, and − 29 ± 3 mV, with Ca^2+^ shifting these values toward less negative potentials (− 9 ± 2, − 15 ± 1, − 25 ± 2, and − 15 ± 5 mV, respectively; Table 1), consistent with partial electrostatic screening of the LPS surface charge.

Electrostatic mapping of human α-thrombin highlights pronounced cationic regions corresponding to exosites 1 and 2 (Fig. 1C), supporting the premise that anionic LPS assemblies can engage thrombin through surface-mediated electrostatic interactions. SDS-PAGE analysis indicated that thrombin remained intact after incubation with various LPS chemotypes, regardless of supramolecular state (micelle or bilayer-like) (Fig. 1D). Thrombin consistently appeared as a predominant band of uniform molecular mass and intensity under all conditions, with no evidence of progressive degradation or discrete cleavage fragments. Minor streaking or faint background signals observed in some lanes were attributed to LPS-related migration artifacts rather than proteolytic activity. These findings indicate that LPS does not induce detectable thrombin proteolysis under the experimental conditions tested.

**Table 1:** Physicochemical properties of LPS aggregates. Critical micelle concentration (CMC), hydrodynamic diameter, polydispersity index (PdI), and zeta potential of LPS aggregates from four different LPS chemotypes, measured in the absence or presence of Ca^2+^. Measurements of diameter, PdI, and ζ-potential were performed at LPS concentrations above the CMC (50 µg/mL). Values are expressed as mean ± SD from *n* = 5 experiments. A dash (–) indicates that CMC was not determined in the presence of Ca^2+^.

LPS	Ca^2+^	CMC (µg/mL)	Diameter (nm)	PdI	ζ-potential (mV)
O111:B4	─	24 ± 1	82 ± 5	0.47 ± 0.1	–13 ± 3
O26:B6	─	18 ± 2	223 ± 14	0.41 ± 0.2	–27 ± 4
*P. aeruginosa*	─	23 ± 4	213 ± 31	0.21 ± 0.1	–35 ± 5
*K. pneumoniae*	─	17 ± 7	92 ± 11	0.55 ± 0.1	–29 ± 3
O111:B4	+	─	129 ± 9	0.45 ± 0.1	–9 ± 2
O26:B6	+	─	342 ± 34	0.54 ± 0.1	–15 ± 1
*P. aeruginosa*	+	─	339 ± 45	0.28 ± 0.2	–25 ± 2
*K. pneumoniae*	+	─	163 ± 14	0.62 ± 0.1	–15 ± 5

### Supramolecular organization of LPS determines binding and inhibition of α-thrombin

**Fig. 2:**
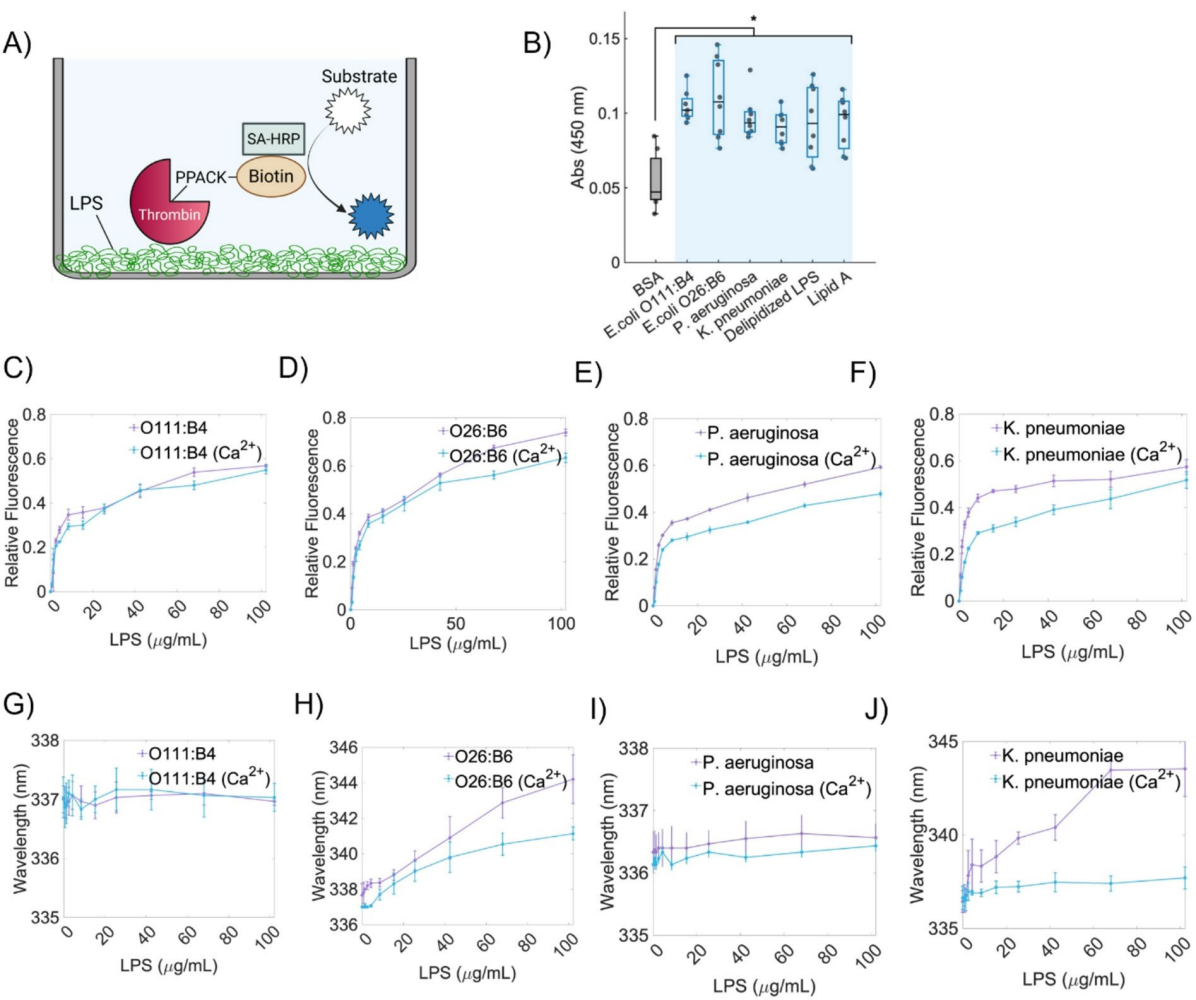
Binding of thrombin to LPS chemotypes. (A–B) Microplate binding assay. LPS chemotypes (and controls) were adsorbed onto microtiter wells (10 µg/well), generating surface-associated LPS coatings, and incubated with PPACK-inhibited, biotinylated thrombin (50 nM) diluted in binding buffer (see Methods). Bound thrombin was detected with SA-HRP/TMB at 450 nm. Statistical significance was determined by one-way ANOVA followed by the Kruskal–Wallis test (*p < 0.05; **p < 0.01; ***p < 0.001). Results are shown as mean ± SEM from three independent experiments. (C–F) Intrinsic fluorescence (quenching) titration. Tryptophan fluorescence of thrombin (0.5 µM) in HEPES-buffered saline was monitored at ~ 336 nm upon titration with increasing concentrations of LPS (3–120 µg/mL). Blue and purple lines represent bilayer-like (+ 10 mM CaCl_2_) and micellar (─ CaCl_2_) LPS structures, respectively. Spectra were normalized from 0 to 1 to facilitate comparison. (G–J) Emission maxima. Wavelengths corresponding to maximum emission are shown for each chemotype. Data represent mean ± SD of three independent measurements.

We assessed direct interactions between human α-thrombin and LPS chemotypes using an ELISA-like binding assay and intrinsic fluorescence spectroscopy (Fig. 2). In the microplate binding assay, all tested LPS preparations supported thrombin binding relative to BSA control (Fig. 2A–B). It is important to note that the LPS immobilized on the microplate surface does not form a continuous planar bilayer but rather adheres as heterogeneous, multilayered or patchy deposits of pre-assembled supramolecular aggregates, particularly when stabilized by Ca^2+^. This surface-associated state preserves key physicochemical features of the LPS aggregates but differs from the native arrangement in bulk solution. Therefore, the assay reflects thrombin interactions with surface-associated LPS assemblies, which is useful for comparative analysis across chemotypes differing in polysaccharide composition and surface charge. All LPS preparations showed higher binding signals than the control, with only modest differences among chemotypes (Fig. 2B). *E. coli* O26:B6 and *E. coli* O111:B4 tended to show slightly higher median signals, whereas *P. aeruginosa*, *K. pneumoniae*, delipidized LPS, and lipid A displayed comparable binding within a similar range. Intrinsic fluorescence titrations, performed in solution with LPS aggregates, showed concentration-dependent changes upon addition of LPS, consistent with rapid thrombin–LPS complex formation (Fig. 2C–F; **Fig. S3**). While the overall interaction appeared largely preserved across supramolecular states, chemotype-dependent spectral shifts (Δλ) revealed that the most pronounced effects involved structural or conformational changes in thrombin rather than differences in binding affinity. Specifically, O26:B6 produced the largest red shift (Δλ ~ 7.5 nm), which remained detectable though reduced under bilayer-like conditions (~ 5.2 nm), whereas *K. pneumoniae* induced a red shift (Δλ ~ 6.9 nm) that was essentially lost upon transition to bilayer-like aggregates (Fig. 2G–J). In contrast, O111:B4 and *P. aeruginosa* showed no detectable Δλ under either supramolecular condition (Fig. 2G, I), indicating minimal conformational perturbation despite binding. We next probed active-site accessibility using p-aminobenzamidine (PABA) fluorescence (**Fig. S4**)^16^. O26:B6 strongly reduced PABA binding, consistent with altered active-site accessibility, whereas *K. pneumoniae* reduced PABA fluorescence only at high, micelle-forming concentrations and not under bilayer-like conditions (**Fig. S4**). Together, these results demonstrate that thrombin binding and conformational/functional outcomes depend on LPS chemotype and supramolecular state, with surface-associated and solution-phase aggregates presenting distinct molecular environments that modulate thrombin interaction.

### Distinct LPS chemotypes differentially regulate thrombin activity and kinetics

**Fig. 3:**
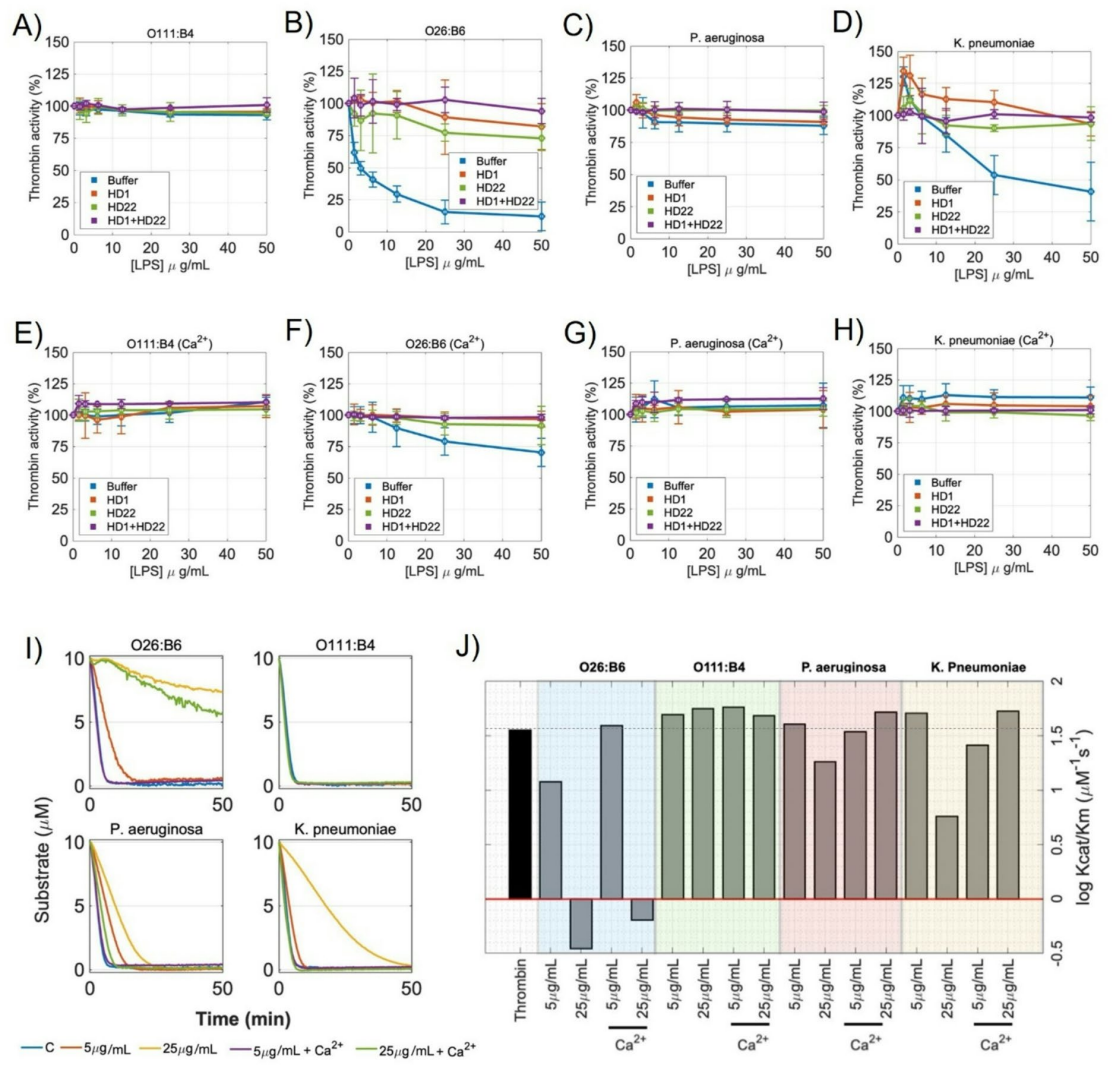
Effect of LPS chemotypes on thrombin enzymatic activity. (A–D) Concentration-dependent inhibition of thrombin activity by LPS chemotypes and restoration by HD-aptamers. The effect on thrombin activity (5 nM) was measured against increasing concentrations of LPS from (A) O111:B4, (B) O26:B6, (C) *P. aeruginosa*, and (D) *K. pneumoniae* in the absence (blue lines) or presence of HD1 (15 µM) (red lines), HD22 (5 µM) (green lines) or HD1 + HD22 (purple lines). Reactions contained S-2238 (200 µM) in HEPES-buffered saline. (E–H) The influence of Ca^2+^-stabilized LPS chemotypes on thrombin activity. Thrombin activity assays were performed as in panels A–D in the presence of LPS prepared with Ca^2+^. (I) Progress curves of S-2238 hydrolysis by thrombin in the presence of LPS chemotypes. Time courses of S-2238 (10 µM) hydrolysis catalyzed by thrombin (0.5 nM) are shown following 15-min pre-incubation with LPS (5–25 µg/mL), prepared with or without Ca^2+^, at 37 °C. (J) Logarithmic values of the catalytic efficiency (log(k_cat_/K_M_)) for S-2238 hydrolysis by thrombin. Experiments were performed in HEPES-buffered saline containing 0.1% PEG 8000 at 37 °C. Data are presented as mean ± SD of experiments performed in triplicate.

We next assessed whether the binding of LPS to thrombin changed the protease activity of the thrombin active site. We measured the initial hydrolysis rates of the chromogenic substrate S-2238 in the presence of increasing concentrations of select LPS chemotypes. We coupled these experiments using aptamers to the thrombin exosites ES1 (HD1, fibrinogen-binding site) and ES2 (HD22, heparin-binding site)^17^, which we predicted should block LPS-thrombin binding (see model in **Fig. S5** generated using PyMOL). LPS from *E. coli* O111:B4 (Fig. 3A) did not alter thrombin activity at any concentration used. In contrast, LPS from *E. coli* O26:B6 (Fig. 3B) and *K. pneumoniae* (Fig. 3D) produced a concentration-dependent inhibition of thrombin activity, while only minimal effects were observed for *P. aeruginosa* LPS (Fig. 3C). The addition of Ca^2+^ reduced the ability of LPS to inhibit thrombin activity, consistent with cation-induced stabilization of LPS supramolecular organization (Fig. 3E–H). We investigated whether thrombin exosites ES1 and ES2, which are known to be highly anionic,^14^serve as binding sites for the polyanionic LPS surface. We used the aptamers HD1 (ES1) and HD22 (ES2) as molecular probes to selectively block these sites on thrombin^17^. We found that masking either exosite partially reduced thrombin inhibition by O26:B6 (Fig. 3B) and *K. pneumoniae* (Fig. 3D) LPS, while simultaneous masking of both exosites nearly eliminated the ability of these LPS chemotypes to inhibit thrombin activity. Following Ca^2+^-mediated stabilization of LPS aggregates, the inhibitory function of LPS from *K. pneumoniae* on thrombin activity was no longer detectable (Fig. 3H), while the inhibitory function of LPS from O26:B6 was partially preserved (Fig. 3F). Similarly to what we observed in the absence of Ca^2+^, the addition of the HD1 and HD22 aptamers eliminated any effects of the LPS chemotypes on thrombin activity in the presence of Ca^2+^ (Fig. 3E–H). Thrombin activity was unaffected by LPS from O111:B4 or *P. aeruginosa*, D-LPS, or isolated lipid A under any condition (Fig. 3A, C and **Fig. S6**).

We next used progress-curve analysis to quantify the kinetics of S-2238 hydrolysis by thrombin and determine how each LPS chemotype alters catalytic parameters (Fig. 3I). Complete reaction traces were fit to the explicit Schnell–Mendoza solution of the integrated Michaelis–Menten equation (see Methods for details), yielding K_M_, V_max_, k_cat_, and k_cat_/K_M_ for each condition. This analysis revealed marked, chemotype-dependent shifts in catalytic efficiency (Fig. 3J). All fitted parameters are reported in **Table ****S1**, and log(k_cat_/K_M_) is summarized in Fig. 3J. We found that LPS from *E. coli* O26:B6 produced a strong, concentration-dependent reduction of thrombin activity. Increasing O26:B6 concentrations led to substantial decreases in k_cat_ and V_max_, with substantial increase in K_M_, resulting in a significant loss of catalytic efficiency. At 25 µg/mL, k_cat_/K_M_ decreased by more than two orders of magnitude compared to thrombin alone. Ca^2+^-mediated stabilization of O26:B6 LPS aggregates partially restored thrombin activity at low LPS concentrations, mainly by recovering k_cat_; however, at higher concentrations, catalytic efficiency remained strongly suppressed, indicating persistent inhibition even in bilayer-like supramolecular states. LPS from *K. pneumoniae* displayed intermediate kinetic behavior. In the absence of Ca^2+^, where LPS is not stabilized as bilayer-like aggregates, catalytic efficiency decreased in a concentration-dependent manner, primarily due to reductions in k_cat_ with comparatively minor changes in K_M_. Ca^2+^-driven reorganization of LPS into more stable aggregates largely restored thrombin activity, with k_cat_/K_M_ returning to near-basal levels, indicating reversible, aggregation-dependent inhibition. In contrast, neither *E. coli* O111:B4 nor *P. aeruginosa* LPS substantially altered thrombin kinetics, including k_cat_ and K_M_, in the presence or absence of Ca^2+^. Overall, these data suggest that LPS binds and modulates the structure and enzymatic activity of the serine protease thrombin in a bacterial chemotype-dependent manner. To investigate the structural diversity of LPS, we analyzed a rough (R-LPS) variant from *E. coli*named as Rd2, characterized by a truncated core and absence of O-specific chains^7,18^. This variant served as a benchmark for comparing thrombin effects across different chemotypes. DLS data demonstrated that Rd2 formed micellar aggregates under Ca^2+^-free conditions, with a hydrodynamic diameter of 201 ± 42 nm. In contrast, pre-assembly with Ca^2+^ resulted in the formation of substantially larger, micron-scale bilayer-like aggregates (3.28 ± 0.39 μm) (**Fig. S7A**). The ζ-potential also shifted from strongly negative values in the absence of Ca^2+^ (−55 ± 3 mV) to less negative values after Ca^2+^ addition (−14 ± 0.5 mV) (**Fig. S7A**). Thrombin activity was evaluated using progress-curve analysis of S-2238 hydrolysis in the presence of Rd2 at concentrations of 5–25 µg/mL (**Fig. S7B**). Relative to control values (K_M_ ~ 1.8 µM; k_cat_ ~ 78.3 s^− 1^), micellar Rd2 increased K_M_ to approximately 3.1 µM at 5 µg/mL and to 24 µM at 25 µg/mL, while k_cat_ shifted to 67 s^− 1^ and 125 s^−^1, respectively (**Fig. S7C**). As a result, catalytic efficiency (k_cat_/K_M_) decreased markedly at 25 µg/mL (~ 8 M^−1^s^− 1^) but remained near control levels at 5 µg/mL. In contrast, Ca^2+^-stabilized Rd2 preparations produced K_M_ and k_cat_ values similar to controls (~ 1.7–2.3 µM and ~ 69–80 s^− 1^), maintaining catalytic efficiencies of approximately 44 and 45.5 M^−1^s^− 1^ at 5 and 25 µg/mL, respectively (**Fig. S7C**).

### LPS chemotypes and supramolecular states differentially regulate fibrin clot formation

**Fig. 4:**
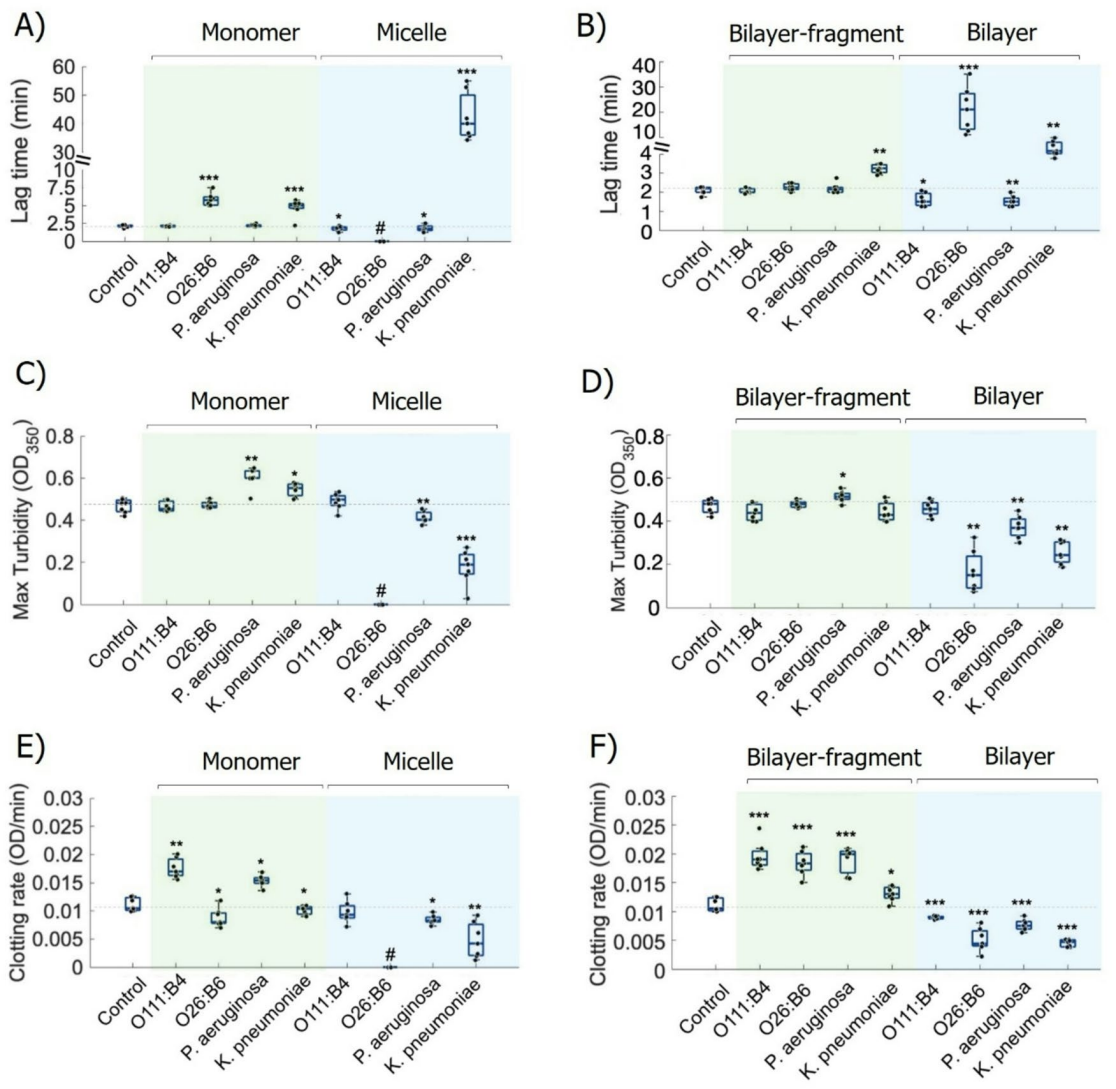
LPS modulation of thrombin-driven fibrin network formation. Effects of different LPS chemotypes on fibrinogen (4 µM) proteolytic clotting triggered by thrombin (2.5 nM) were quantified in terms of lag time (A, B), maximum turbidity (C, D), and clotting rate (E, F). LPS were tested in two concentration regimes and structural forms: 5 µg/mL (monomers or bilayer-fragments formed in the presence of Ca^2+^) and 25 µg/mL (micelles or bilayers formed in the presence of Ca^2+^). Data are presented as mean ± SD of triplicate experiments. Statistical analysis was performed using non-parametric one-way ANOVA followed by the Kruskal–Wallis test (*p < 0.05, **p < 0.01, ***p < 0.001 vs. control).

The potential for LPS-modulated thrombin activity to alter fibrin formation was assessed using a purified fibrinogen system (Fig. 4; **Fig. S8**). Turbidimetry, which measures changes in solution cloudiness as fibrin forms, revealed pronounced, chemotype-specific effects on fibrin polymerization (Fig. 4A–F; **Fig. S8**). *E. coli* O26:B6 demonstrated a unique, state-dependent profile; while O26:B6 micelles completely abolished clot formation (indicated by #, Fig. 4A, C, E), this inhibitory effect was reversed or significantly attenuated when the LPS was prepared as bilayer-like aggregates, which resulted in fibrin clot formation (Fig. 4B, D, F), allowing a transition to fibrin clot formation. In contrast, *K. pneumoniae* also impaired clotting in the purified fibrinogen system, but its effect was primarily kinetic and more variable than that of O26:B6. Micellar *K. pneumoniae* preparations increased lag time (~ 50 min in the micelle condition; Fig. 4A) and reduced final clot density, but did not completely prevent fibrin clot formation. In contrast, *E. coli* O111:B4 and *P. aeruginosa* primarily increased the rate of fibrin assembly. These chemotypes consistently accelerated the fibrin growth phase, particularly in bilayer-fragment states, where they significantly increased the clotting rate (Fig. 4F) and either maintained or slightly enhanced maximum turbidity. Overall, these results demonstrate that LPS-driven modulation of fibrin formation is not universal. The effects range from complete kinetic suppression (O26:B6) to enhanced assembly rates (O111:B4 and *P. aeruginosa*), depending on the specific LPS chemotype and its supramolecular organization.

**Fig. 5:**
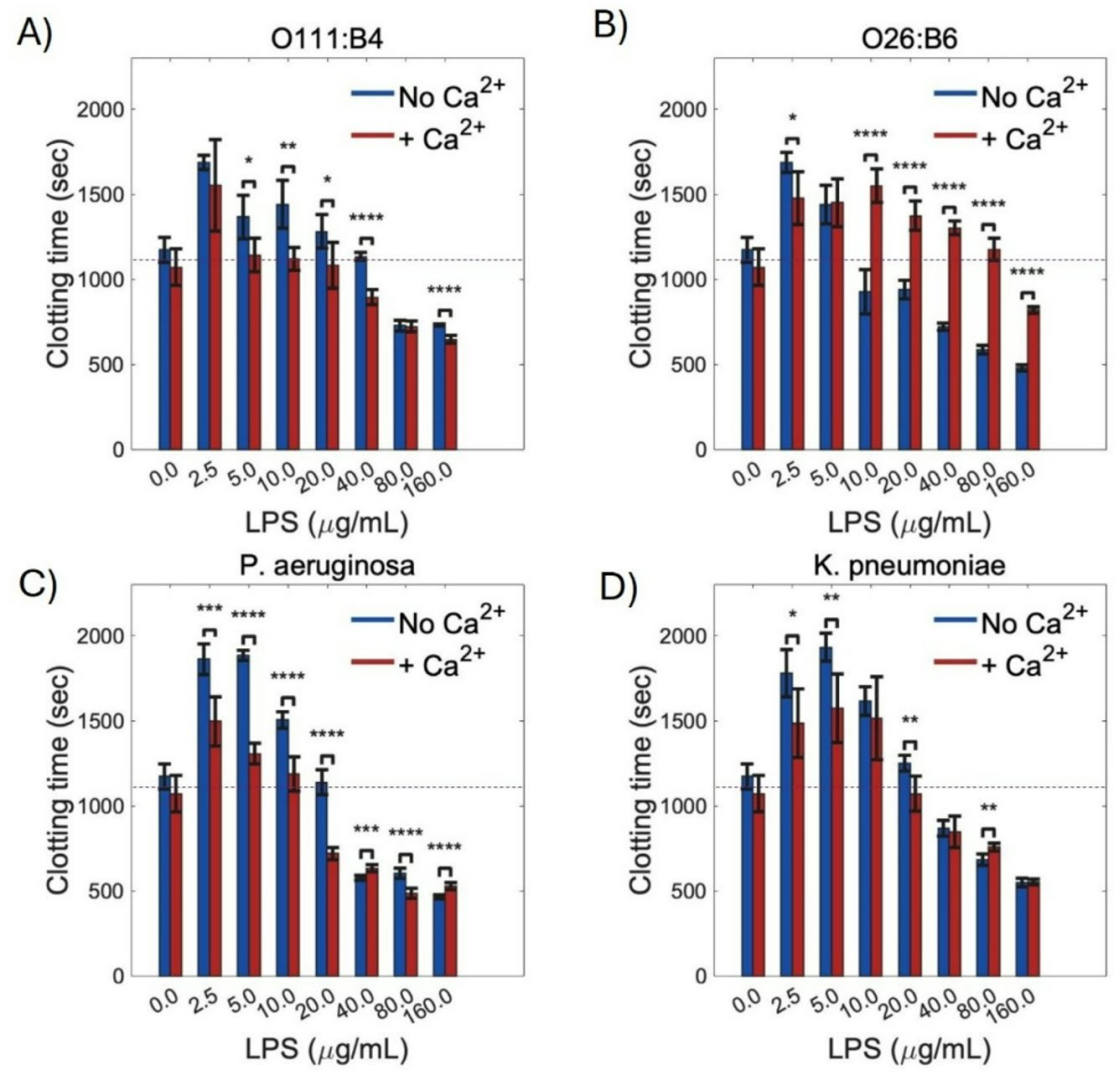
Clotting time of recalcified PPP in the presence of LPS chemotypes. Citrated human plasma was incubated with increasing concentrations of LPS from *E. coli* O111:B4 (A), *E. coli* O26:B6 (B), *P. aeruginosa* (C), and *K. pneumoniae* (D) prepared either in the absence (blue) or presence (red) of Ca^2+^. Clotting time was determined using a coagulation analyzer. Plasma alone coagulated after approximately 1000 s, serving as the baseline. At low concentrations, some LPS preparations slightly prolonged clotting time, whereas higher concentrations produced marked acceleration of coagulation. The presence of Ca^2+^ significantly enhanced the procoagulant activity of O111:B4 and *P. aeruginosa* LPS, while O26:B6 exhibited reduced activity upon Ca^2+^ addition. *K. pneumoniae* LPS displayed an intermediate response. Data represent mean ± SD from ten independent experiments (*n* = 10). Statistical significance was assessed by comparing Ca^2+^-free versus Ca^2+^-containing LPS at each concentration using non-parametric one-way ANOVA followed by the Kruskal–Wallis test (*p < 0.05, **p < 0.01, ***p < 0.001, ****p < 0.0001).

To evaluate the effect of LPS on coagulation, we tested thrombin-induced clotting in recalcified platelet-poor plasma (PPP) using LPS pre-assembled as either micelles or bilayer-like aggregates. First, we validated the colloidal stability of LPS aggregates in blood plasma, noting a heterogeneous population of large and small assemblies (**Fig. S9**). This implies a balance between protein-induced disaggregation and the formation of a biomolecular corona, as previously reported^6^. After mixing the LPS with PPP, CaCl_2_ (6 mM) was added solely for recalcification to initiate coagulation and was not intended to modulate the LPS assembly state. Under these conditions, *E. coli* O111:B4 and *P. aeruginosa* displayed a biphasic concentration-dependent pattern (Fig. 5A, C): lower concentrations (~ 2.5–10 µg/mL) were associated with delayed clot initiation, whereas higher concentrations (≥ 20 µg/mL) shifted the response toward faster clotting, as indicated by shorter clotting times. This procoagulant shift was preserved, and in some conditions appeared more pronounced, when LPS was pre-assembled in the presence of Ca^2+^. *K. pneumoniae* showed a similar overall directional trend (Fig. 5D), with delayed clot initiation at lower concentrations and shorter clotting times at higher concentrations; however, the magnitude of this effect was more modest and more variable than that observed for O26:B6. In contrast, O26:B6 exhibited a distinct supramolecular-state-dependent profile (Fig. 5B). Although both preparations delayed clotting at lower concentrations, their behaviors diverged at higher concentrations, with micellar O26:B6 transitioning to shorter clotting times above 10 µg/mL, whereas O26:B6 pre-assembled with Ca^2+^ sustained prolonged clotting times across the same concentration range and preserved the delayed-clotting phenotype even at 160 µg/mL. Collectively, these results indicate that both LPS chemotype and pre-defined supramolecular organization are key determinants of fibrin formation kinetics in plasma, and that the concentration range associated with delayed versus accelerated clotting differs across chemotypes.

## Discussion

Endotoxemia, characterized by the presence of bacterial LPS in circulation, is a well-established driver of thromboinflammation and coagulation activation, contributing to systemic inflammatory syndromes^19,20^. While LPS is known to trigger upstream coagulation pathways such as the contact pathway^6^, its direct effects on thrombin, particularly in the context of LPS structural heterogeneity and supramolecular organization, remain incompletely understood. Here, we show that LPS chemotype and assembly state are key determinants of thrombin regulation, influencing binding, conformational dynamics, and functional outcomes.

Previous work has shown that certain LPS chemotypes can modulate thrombin activity in purified systems^21^. We extend these observations by highlighting the role of LPS supramolecular organization in shaping thrombin function. The critical role of LPS aggregation is consistent with established biophysical principles wherein hydrophobic lipid A self-association competes with electrostatic repulsion from anionic phosphate groups, defining the critical micelle concentration and higher-order assembly^22–24^. In this context, divalent cations such as Ca^2+^promote more ordered aggregates by bridging phosphate groups and reducing electrostatic repulsion^4,6,9,25–27^. Although the Ca^2+^concentrations used here exceed physiological levels, they provide a controlled framework to stabilize LPS structures. Distinct LPS chemotypes, differing in polysaccharide length, phosphate content, and acylation patterns, display distinct supramolecular behaviors that influence thrombin interaction^3,5,8^. For example, *E. coli* O111:B4 (S-LPS) is known to form relatively stable aggregates with reduced surface negativity due to Ca^2+^-mediated screening, ^28,29^ whereas *P. aeruginosa* (S-LPS) has been reported to exhibit more negative ζ-potentials, consistent with its highly phosphorylated core. ^30,31^ Similarly, SR-LPS chemotypes such as *E. coli* O26:B6 and *K. pneumoniae*, characterized by shorter polysaccharide chains and comparable phosphate content, are generally associated with highly anionic assemblies in the absence of Ca^2+^and undergo a shift toward larger, less negatively charged aggregates upon cation-mediated stabilization^32–34^. Such reorganization is expected to enhance electrostatic interactions with thrombin exosites, although subtle differences in their core or O-antigen composition may influence the degree of thrombin modulation. Together, these features underscore the importance of LPS molecular architecture in regulating thrombin function.

Functionally, thrombin binding profiles appear strictly dependent on the integrity of the LPS molecule. The observation that intact LPS binds thrombin more effectively than detoxified versions or isolated lipid A suggests that the complete molecular architecture is required to stabilize the complex. Furthermore, the physical state of these molecules is a primary determinant of function; micelle-forming LPS induces more pronounced conformational changes in thrombin compared to bilayer-like aggregates. This indicates that thrombin is highly sensitive to the curvature and charge density of the interacting surface^35–37^. In a solid-phase context, where LPS forms multilayered deposits, these structures retain key features of free-floating aggregates while providing a stable platform for protein interaction, effectively linking the biochemical requirement for the polysaccharide chain to the structural context of the bacterial surface.

The interaction between LPS and thrombin’s exosites (ES1 and ES2) points toward a model of allosteric inhibition. The fact that blocking both exosites abolishes LPS-induced inhibition suggests that LPS binding outside the active site triggers conformational shifts that impair catalytic efficiency. This aligns with the broader understanding of how exosite engagement governs thrombin substrate specificity^11,17,38^. Interestingly, the correlation between the extent of inhibition and the supramolecular state, where micelles exert stronger effects than bilayers, highlights a hierarchy of inhibitory potential. The finding that even deep-rough chemotypes lacking extended O-antigens^18^can modulate activity reinforces the idea that the lipid A/core region is the fundamental engine of this interaction, though its effects are refined by the rest of the molecule^6,39^..

The divergence between chromogenic activity and fibrin clot formation reveals the complexity of these interactions. While chromogenic substrates measure active-site cleavage^40^,fibrin formation is a multi-step process involving exosite-mediated binding and polymerization^11,14,38^. The potent inhibition of fibrin formation by certain micellar LPS chemotypes, compared to the mere delay caused by their bilayer-like counterparts, suggests that the physical assembly of LPS can physically or allosterically shield thrombin from its bulkier natural substrates. In a plasma environment, the integrated clotting phenotype reflects these competing influences^42–44^. The biphasic, concentration-dependent effects of LPS supramolecular states on clotting times emphasize that the nature of coagulation dysregulation in sepsis is not uniform but is governed by the specific “fingerprint” of the circulating LPS chemotype^4,6^..

A key finding of our study is the similar behavior of the SR-LPS chemotypes from *E. coli* O26:B6 and *K. pneumoniae*. Their reduced O-antigen length increases the exposure of anionic phosphate groups, promoting electrostatic interactions with thrombin’s cationic regions. The differences observed between these LPS variants likely arise from variations in phosphate group number and accessibility, as well as in the composition and organization of their saccharide moieties^28,33^. These features can influence local charge distribution, hydration, and protein-binding properties. We propose that this results in two complementary mechanisms: an allosteric effect, in which binding reduces catalytic activity, and a steric effect, in which larger LPS assemblies hinder access to bulky substrates such as fibrinogen. Together, this framework explains how structurally related chemotypes from different bacteria can produce similar inhibitory profiles and highlights the balance between core exposure and O-antigen length as a key determinant of host protein modulation.

**Fig. 6:**
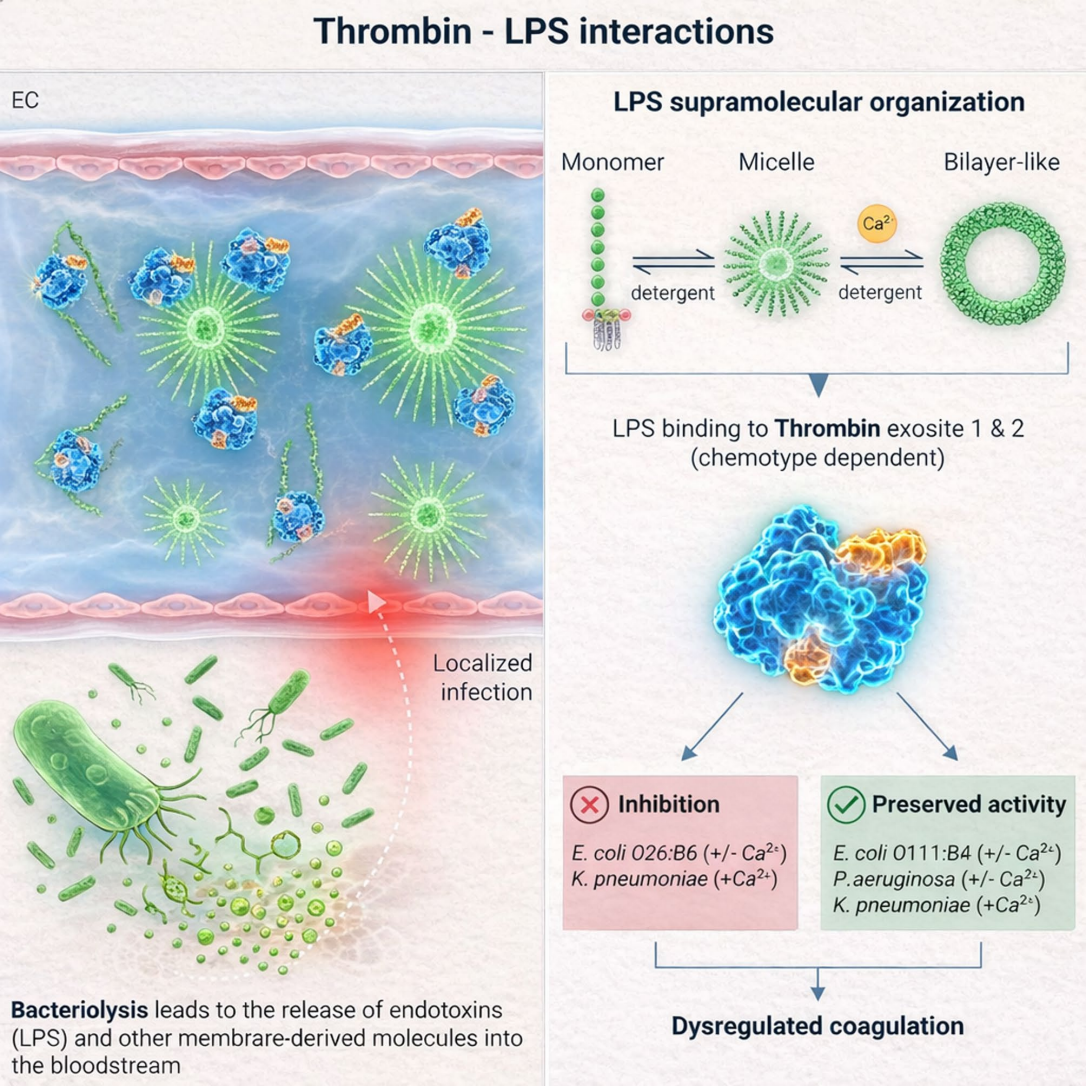
Thrombin–LPS interactions and the role of LPS supramolecular organization in coagulation. *Left panel*: During localized Gram-negative bacterial infection, bacteriolysis releases lipopolysaccharides (LPS, endotoxins) and other membrane-derived molecules into the extracellular milieu and potentially into the bloodstream. LPS released during bacteriolysis can cross the endothelial barrier and enter the vascular lumen, where supramolecular assemblies existing as monomers, micelles, or bilayer-like aggregates come into contact with plasma components and directly interact with coagulation factors, including thrombin. *Right panel*: LPS supramolecular organization and chemotype influence thrombin activity. LPS transitions between monomeric, micellar, and bilayer-like states are modulated by detergents and Ca^2+^. LPS binds thrombin ES1 and ES2 in a chemotype-dependent manner, leading to either inhibition (e.g., *E. coli* O26:B6; *K. pneumoniae*) or preserved activity (e.g., *E. coli* O111:B4; *P. aeruginosa*; *K. pneumoniae*) depending on Ca^2+^ availability. These interactions may contribute to dysregulated coagulation during Gram-negative infections by delaying fibrin formation, interfering with cofactor binding, and altering fibrin network architecture. EC, endothelial cells; LPS, lipopolysaccharide; Ca^2+^, calcium ion.

Overall, this study supports a framework in which LPS chemotype-specific physicochemical properties and supramolecular plasticity modulate thrombin exosite interactions, allosteric regulation, and downstream clotting outcomes, as illustrated in the conceptual model presented in Fig. 6. In this framework, LPS supramolecular state and Ca^2+^ availability collectively shape thrombin–LPS interactions, helping explain why distinct chemotypes produce divergent effects on thrombin activity and fibrin formation. These mechanistic insights advance our understanding of how LPS heterogeneity may contribute to variable coagulation responses in sepsis and systemic inflammation. Future studies should extend this framework to a broader range of LPS variants and in vivo models to further define the physiological relevance and therapeutic implications of LPS–thrombin interactions.

## Materials and methods

**Reagents.** Lipopolysaccharides from *Escherichia coli* O111:B4 (#L2630), O26:B6 (#L8274), and F583 (Rd2 mutant) (#L6893), *Pseudomonas aeruginosa* (#L9143), and *Klebsiella pneumoniae* (#4268), as well as detoxified LPS (D-LPS, #L3023), were purchased from Sigma-Aldrich. Lipid A was obtained from Avanti Polar Lipids. Human α-thrombin was purchased from Prolytix, and fibrinogen was obtained from Enzyme Research Laboratories. The chromogenic thrombin substrate S-2238 (H-D-Phe-Pip-Arg-pNA) was from Chromogenix. Aptamers HD1 and HD22 were synthesized by Integrated DNA Technologies (IDT). N-phenyl-1-naphthylamine (NPN), *p*-aminobenzamidine (PABA), bovine serum albumin (BSA), dithiothreitol (DTT), PPACK (D-Phenylalanyl-L-prolyl-L-arginine chloromethyl ketone), and all buffer components including HEPES, NaCl, CaCl_2_, NaHCO_3_, KCl, dextrose, and PEG 8000 were purchased from Sigma-Aldrich unless otherwise specified. Streptavidin–horseradish peroxidase (HRP) conjugate was obtained from Invitrogen, and 3,3′,5,5′-tetramethylbenzidine (TMB) substrate from ThermoScientific (Pierce). Citrated human platelet-poor plasma was prepared in-house from healthy donors as described below.

### Computational representation of LPS chemotypes

 LPS chemotype molecules from *E. coli* chemotypes O111:B4 and O26:B6, *K. pneumoniae* O1-1, and *P. aeruginosa* O10 were generated using CHARMM-GUI (http://charmm-gui.org/)^45,46^ and subsequently visualized and processed with PyMOL. LPS molecules were represented as amphiphilic assemblies, and micellar *versus* bilayer-like arrangements were depicted as schematic models under monovalent *versus* divalent ionic conditions. These models are intended for visualization/conceptual illustration and were not subjected to molecular dynamics simulations or experimental validation of stability.

### CMC determination of LPS via NPN fluorescence assay

 The critical micelle concentration (CMC) of LPS from *E. coli* O111:B4, *E. coli* O26:B6, *P. aeruginosa*, and *K. pneumoniae*was using N-Phenyl-1-naphthylamine (NPN), a polarity-sensitive fluorescent probe that increases in fluorescence intensity upon partitioning into hydrophobic environments^4,22^. Measurements were conducted at 25 °C in HEPES-buffered saline (20 mM HEPES pH 7.4, 150 mM NaCl), using a CARY Eclipse Fluorescence Spectrophotometer with a quartz cuvette. NPN exhibits a low quantum yield in aqueous environments, with an emission maximum near 475 nm. When partitioned into the hydrophobic interior of LPS aggregates, its fluorescence intensity increases significantly, and the emission spectrum shifts to approximately 425 nm. This characteristic was used to monitor micelle formation. LPS was titrated across concentrations ranging from 0 to 100 µg/mL. Each sample contained a fixed NPN concentration (5 µM), prepared from a 2.5 mM stock solution. Samples were equilibrated for 30 min at 25 °C prior to analysis. Fluorescence emission was recorded at 425 nm following excitation at 350 nm, with both excitation and emission slit widths set to 5 nm. The addition of CaCl_2_ induced the aggregation and cross-linking of LPS molecules, and as a consequence, prevented the calculation of CMCs for LPS in the presence of Ca^2+^.^4,22–24^

### LPS aggregates preparation

 Suspensions of LPS aggregates were prepared by dissolving LPS from *E. coli* (O111:B4 and O26:B6), *K. pneumoniae*, and *P. aeruginosa* at 1 mg/mL in HEPES-buffered saline (20 mM HEPES, pH 7.4, 150 mM NaCl), either in the absence or presence of 10 mM CaCl_2_. Each sample was vortexed vigorously for 10 min, followed by sonication for 30 min in a 60 °C water bath. Afterwards, samples underwent multiple thermal shock cycles by alternating incubation between 4 °C and 60 °C. To ensure equilibration, the suspensions were incubated at 4 °C for at least 12 h before use in experiments. LPS samples prepared with 10 mM CaCl_2_ are referred to throughout the manuscript as “Ca^2+^-stabilized LPS.” The inclusion of Ca^2+^ during this pre-assembly step induces the formation of stable, bilayer-like LPS aggregates, thereby establishing defined supramolecular states prior to downstream functional assays.

### Hydrodynamic size and surface charge characterization of LPS aggregates

The hydrodynamic size distribution and zeta potential of LPS aggregates, prepared with or without CaCl_2_(10 mM), were characterized using dynamic light scattering (DLS) and laser Doppler microelectrophoresis, respectively, according to previously reported methods^6^. Both analyses were conducted on a Malvern Zetasizer Nano ZS instrument (Malvern Panalytical, Westborough, MA, USA) maintained at 25 °C. For DLS measurements, LPS samples were diluted to a final concentration of 50 µg/mL in HEPES buffer (20 mM HEPES pH 7.4). The intensity of scattered light was recorded at a backscatter angle of 173° using a 4 mW He-Ne laser (633 nm). For each sample, ten consecutive measurements (approximately 100 runs total) were acquired. All experiments utilized five independent samples. The resulting intensity autocorrelation functions were deconvoluted using the instrument’s CONTIN algorithm to obtain distributions of diffusion coefficients^47^. These values were converted into hydrodynamic diameters (d_h_) using the Stokes-Einstein equation. The polydispersity index (PdI) was reported to indicate the width of the size distribution. To assess the influence of a complex biological medium on LPS aggregation, DLS was used to monitor LPS dynamics in diluted human plasma. Test samples were prepared by incubating the same concentration of LPS in a solution of 10% (v/v) citrated human platelet-poor plasma (PPP) in HEPES buffer for 10 min. DLS measurements for both control and PPP-containing samples were performed after this incubation period to enable direct comparison of aggregate size distributions. To correlate aggregate size with surface charge, the zeta (ζ)-potential of the same LPS preparations (50 µg/mL) in HEPES buffer, with or without CaCl_2_(10 mM) was investigated under identical sample conditions. Electrophoretic mobility was determined by laser Doppler anemometry at a 90° scattering angle. A refractive index of 1.330 and a viscosity of 0.8872 cP were applied for the dispersant. The ζ-potential was calculated from the mean electrophoretic mobility (10 measurements, ~ 50 runs each) using the Smoluchowski model^48^..

### Electrostatic calculations of α-thrombin

 The structures of human α-thrombin (dₕ~5 nm, 37 kDa)^13,49^(PDB: 1PPB) and thrombin in complex with the aptamers HD1 (PDB: 6Z8X), HD22 (PDB: 8TQS), and HD1 + HD22 (PDB: 5EW1) were obtained from the Protein Data Bank (https://www.rcsb.org). Electrostatic surface potentials were calculated at pH 7.4 and 150 mM NaCl using the Adaptive Poisson–Boltzmann Solver (APBS). All structures were processed and visualized with PyMOL^50^..

### Assessment of α-thrombin integrity following LPS exposure

The stability of human α-thrombin after LPS treatment was evaluated using SDS–PAGE. Samples containing human α-thrombin (3 µM) were incubated for 15 min at 37 °C with or without LPS (200 µg/mL) derived from *E. coli* (O111:B4 and O26:B6), *K. pneumoniae*, and *P. aeruginosa* in a total volume of 100 µL. Following incubation, proteins were denatured by boiling after combining 20 µL of the LPS-treated thrombin sample with 20 µL of gel loading buffer. The samples were then resolved on 10% polyacrylamide gels under denaturing conditions.

### ELISA-like binding assay

Thrombin–LPS binding was quantified using an ELISA-like assay^51^ as schematized in Fig. 2A. LPS-coated wells represent surface-associated immobilized LPS assemblies rather than a continuous planar membrane. Various LPS chemotypes, lipid A, and D-LPS were immobilized at 10 µg/well in microtiter plates (Corning) by incubation in 0.1 M sodium carbonate coating buffer (pH 9.6) overnight at 4 °C. After immobilization, wells were blocked for 1 h at 37 °C with 1% BSA to minimize non-specific binding. Wells were then washed and incubated for 1 h at 37 °C with human α-thrombin (50 nM), which was biotinylated and active-site blocked with PPACK. The modified thrombin was diluted in modified HEPES-buffered saline (12 mM HEPES pH 7.1, 150 mM NaCl, 12 mM NaHCO_3_, 3 mM KCl, 5.5 mM dextrose) supplemented with 0.1% BSA. Bound biotinylated thrombin was detected using a HRP conjugate and the peroxidase substrate 3,3’,5,5’-tetramethylbenzidine. The reaction was performed according to the manufacturer’s instructions. Absorbance of the reaction product was measured at 450 nm using a Tecan microplate reader to quantify thrombin binding.

### Intrinsic fluorescence spectroscopy

Thrombin–LPS interactions were assessed by monitoring intrinsic fluorescence of thrombin during titration with increasing LPS concentrations using a CARY Eclipse Fluorescence Spectrophotometer and a quartz cuvette at 25 °C. Intrinsic fluorescence primarily reflects local environmental and conformational changes around aromatic residues (Trp/Tyr)^52^. Complex formation between α-thrombin and LPS was analyzed by fluorescence quenching titration measurements. Intrinsic tryptophan fluorescence spectra of native α-thrombin were recorded using 0.5 µM protein diluted in HEPES-buffered saline. Interactions with LPS were evaluated by titrating the protein with increasing LPS concentrations up to 120 µg/mL. The intrinsic Trp was excited at 280 nm, and emission was monitored from 300 to 500 nm with a slit width of 5 nm. The internal filter effect from LPS was corrected by titrating a solution of the amino acid Trp with identical concentrations of LPS. Corrected quenching curves for the native proteins were generated by dividing the uncorrected data by the Trp reference curve. Emission intensities at approximately 336 nm were recorded one minute after each titration point and plotted. A skewed Gaussian function was applied to determine the wavelengths of maximum emission. Emission spectra were recorded and both fluorescence intensity changes (complex formation) and spectral shifts (Δλ; changes in emission maximum) were quantified.

### Effect of LPS on thrombin active-site accessibility

Active-site accessibility was evaluated using p-aminobenzamidine (PABA), a fluorescent ligand whose emission increases upon binding to the thrombin active site^16^. Thrombin active-site accessibility was assessed using fluorescence assays conducted in black 96-well plates (Corning). The assays included α-thrombin (50 nM), PABA (50 µM), and PPACK (500 nM) in the presence of LPS chemotypes (5–25 µg/mL) prepared with or without CaCl_2_ (10 mM). Thrombin, LPS, PPACK, and their combinations were incubated for 15 min at 37 °C. Subsequently, PABA (50 µM) was added to all samples, including controls. After an additional 15-minute incubation at 37 °C, fluorescence was measured using a Tecan plate reader (excitation 345 nm, emission 370 nm).

### Thrombin amidolytic activity

Thrombin activity was quantified using the chromogenic tripeptide substrate S-2238 in 96-well microtiter plates (Corning). Reactions were performed in HEPES-buffered saline supplemented with 0.1% PEG 8000, and absorbance was recorded at 405 nm using a Tecan microplate reader maintained at 37 °C. Increasing concentrations of LPS chemotypes (0–50 µg/mL) were incubated with human α-thrombin (5 nM final concentration) in assay buffer. To evaluate supramolecular-state dependence, LPS was used either as micelle or as bilayer-like preparations generated by Ca^2+^ pre-assembly as described in “LPS aggregates preparation”. Unless otherwise stated, no additional CaCl_2_ was introduced as an experimental variable during the chromogenic measurements beyond what was present from the LPS preparation step. Reactions were initiated by simultaneous addition of S-2238 (200 µM final concentration) to all wells. Control wells containing LPS plus substrate without thrombin were included for background correction. Substrate hydrolysis was monitored by recording A_405_ at 15 s intervals for 30 min. Initial velocities (*v*_*0*_) were calculated from the slope of the initial linear phase of the progress curves, and relative activity was expressed as *v*_*0*_ in the presence of LPS normalized to *v*_*0*_in buffer alone. For competition assays probing thrombin exosites, α-thrombin was co-incubated with LPS chemotypes and the DNA aptamers HD1 (exosite 1) and/or HD22 (exosite 2) prior to substrate addition. Aptamers were used at final concentrations of 15 µM (HD1) and 5 µM (HD22), either individually or in combination^35,36^..

### Kinetic analysis of thrombin activity

 Catalytic parameters of human α-thrombin in the absence or presence of LPS were determined by progress-curve analysis. Thrombin-catalyzed cleavage of the chromogenic substrate S-2238 was monitored continuously by recording the increase in absorbance at 405 nm for 60 min at 37 °C using a Tecan plate reader. Rather than estimating initial velocities, complete reaction traces were analyzed using the explicit Schnell–Mendoza solution to the integrated Michaelis–Menten equation under the quasi–steady-state assumption (QSSA):

1 $$\:\left[\mathrm{S}\right]\left(\mathrm{t}\right)={\mathrm{K}}_{\mathrm{M}}\times\:\mathrm{W}\left[\frac{{\left[\mathrm{S}\right]}_{0}}{{\mathrm{K}}_{\mathrm{M}}}\mathrm{e}\mathrm{x}\mathrm{p}\left(\frac{{(-\mathrm{V}}_{\mathrm{m}\mathrm{a}\mathrm{x}}\mathrm{t})+[{\mathrm{S}}_{0}]}{{\mathrm{K}}_{\mathrm{M}}}\right)\right]\:$$

where [S] is the substrate concentration at time t, [S_0_] is the initial substrate concentration, K_M_ is the Michaelis constant, and V_max_ is the maximum reaction velocity. W denotes the Lambert-W (Omega) function, defined by W(x)exp[*W*(x)] = x^53,54,55^. In this model, the asymptotic behavior of W(x) can be approximated as W(x) ~ ln(x) – ln(ln(x)). To assess LPS effects on thrombin kinetics, human α-thrombin (0.5 nM) was incubated with LPS at 5–25 µg/mL for 15 min at 37 °C in HEPES-buffered saline. LPS preparations were generated either without Ca^2+^ (micelle-forming condition) or with Ca^2+^ during pre-assembly (bilayer-like/Ca²⁺-stabilized condition) as described above. No additional CaCl_2_ was added to the kinetic assay buffer; thus, Ca^2+^ was used only during the LPS pre-assembly step to define the supramolecular state. Reactions were initiated by addition of S-2238 (final 10 µM), and A405 was recorded over time. Absorbance traces were converted to product concentration [P](t) using the Beer–Lambert law ([P] = A_405_/(ε)∙ *l*), where $\:{\upepsilon\:}$ is the molar extinction coefficient of p-nitroaniline at 405 nm (ε ~ 9600 M^−1^cm^− 1^) and *l* is the effective pathlength of the microplate well (~ 0.3 cm). Progress curves were fit by nonlinear regression to Eq. 1 to estimate K_M_​ and V_max_. k_cat_ was calculated as V_max_/[E]_total_, and catalytic efficiency was calculated as k_cat_/K_M_​.

### Fibrin clot formation by optical turbidimetry

 Turbidity assays were performed using a Tecan microplate reader. α-Thrombin (2.5 nM) was pre-incubated with LPS chemotypes at concentrations of 5–25 µg/mL, previously prepared, either without or with CaCl_2_, for 15 min at 37 °C in a 96-well plate. Clotting was initiated by the addition of fibrinogen (4 µM) in HEPES-buffered saline, and fibrin formation was monitored at 37 °C by measuring turbidity at 350 nm. Control wells without LPS were included. All concentrations are reported as final values after mixing. Turbidity progress curves were analyzed using the “Clotting_or_HaloCL” Shiny application to extract lag time, defined as the time to reach 5% of maximum turbidity, and maximum turbidity^56,57^. Clotting rate was determined in Python by normalizing each curve to its plateau and fitting the linear segment immediately following the lag phase to obtain the slope.

### Plasma clotting assays

 All experiments involving human participants were approved by the Oregon Health & Science University Institutional Review Board (IRB #1673). All methods were performed in accordance with the relevant guidelines and regulations. Venous blood from healthy adult volunteers of both sexes was collected by standard venipuncture into tubes containing sodium citrate (0.32% w/v), and written informed consent was obtained from each donor. Platelet-poor plasma (PPP) was prepared by centrifuging citrated whole blood at 2150 × g for 10 min, followed by a second centrifugation under identical conditions to minimize platelet contamination. PPP from three independent donors was pooled and stored at − 80 °C until use. Clotting times were measured using a KC4 Coagulation Analyzer (Trinity Biotech PLC, Bray, Ireland). PPP was diluted threefold in HEPES-buffered saline to standardize ionic conditions and reduce matrix effects. The diluted PPP was mixed in coagulometer cuvettes and incubated for 5 min at 37 °C with LPS chemotypes previously prepared, either without or with Ca^2+^, to promote distinct supramolecular assemblies and facilitate initial interactions with plasma proteins. Clotting was initiated by recalcification with CaCl_2_ to a final concentration of 6 mM. HEPES-buffered saline without LPS was used as vehicle control.

### Data analysis

 Analyses were conducted in Python utilizing custom scripts and standard scientific libraries. Bar plots present the mean with 95% confidence intervals. Time-course plots display the mean, with 95% confidence intervals when specified. Boxplots indicate the median and interquartile range, with notches representing the median’s 95% confidence interval and whiskers extending to 1.5 times the interquartile range. Pairwise comparisons between independent groups were performed using two-sided Mann–Whitney U tests. Statistical significance was denoted as follows: *p* < 0.05 (*), *p* < 0.01 (**), *p* < 0.001 (***), and *p* < 0.0001 (****). Assumptions of normality and equal variances were not required, but independence of observations was assumed.

## Supplementary Information

Below is the link to the electronic supplementary material.

Supplementary Material 1

## Data Availability

All data described in this work are included in the manuscript and the supporting materials. Further information can be obtained from the corresponding author upon reasonable request.
