# Supplementary material for: Molecular basis for inhibition of α-thrombin activity by bacterial lipopolysaccharides
Source: Sci Rep. 2026 Mar 27;16:10851. doi: 10.1038/s41598-026-46276-5 (PMC13040036; doi:10.1038/s41598-026-46276-5)
Supplement: Supplementary file 1 — Supplementary Material 1 [file 41598_2026_46276_MOESM1_ESM.docx]

**Supporting information**

**Molecular basis for inhibition of α-thrombin activity by bacterial lipopolysaccharides**

André L. Lira^1*^, Katelyn C. Drew^1^, Rodrigo L. M. Dantas^2^, Jiaqing Pang^1^, Owen J. T. McCarty^1^

^1^Department of Biomedical Engineering, Oregon Health & Science University, Portland, 97239, USA

^2^Department of Science and Technology, Federal University of São Paulo, São José dos Campos, 12245-000, Brazil

^*^Corresponding author: dasilva@ohsu.edu

**List of contents:**

**Table S1.** *Kinetic parameters of α-thrombin in the absence or presence of bacterial LPS* (S-2)

**Figure S1.** *Critical micelle concentration (CMC) of LPS chemotypes* (S-3)

**Figure S2.** *Size distribution of LPS chemotype aggregates* (S-4)

**Figure S3.** *Quenching of thrombin intrinsic fluorescence by LPS* (S-5)

**Figure S4.** *LPS effect on PABA binding to thrombin* (S-6)

**Figure S5.** *Thrombin–aptamer electrostatic surface* (S-7)

**Figure S6.** *Concentration-dependent inhibition of thrombin by D-LPS and lipid A* (S-8)

**Figure S7.** *Figure S7. Physicochemical characterization of rough LPS (Rd2) and its effects on thrombin progress-curve* (S-9)

**Figure S8.** *Time course of fibrin clot formation by turbidity measurements* (S-10)

**Figure S9.** *Size distribution of LPS in human plasma* (S-11)

|  | [LPS]  (μg/mL) | Ca^2+^ | *K_M_*  (μM) | 10^-2^ × *V_max_*  (μM/s) | *K_cat_*  (1/s) | *K_cat_*/*K_M_*  (1/(μM·s)) |
| --- | --- | --- | --- | --- | --- | --- |
| Thrombin | ─ | ─ | 1.8 ± 0.8 | 3.9 ± 0.01 | 78 ± 3 | 43 ± 3 |
| O111:B4 | 5 | ─ | 1.6 ± 0.3 | 4.1 ± 0.01 | 81 ± 3 | 50 ± 8 |
|  | 25 | ─ | 1.3 ± 0.2 | 3.8 ± 0.01 | 76 ± 3 | 56 ± 10 |
|  | 5 | + | 1.3 ± 0.2 | 3.7 ± 0.01 | 75 ± 2 | 58 ± 11 |
|  | 25 | + | 1.7 ± 0.3 | 4.2 ± 0.02 | 83 ± 4 | 48 ± 8 |
| O26:B6 | 5 | ─ | 2.9 ± 0.5 | 1.7 ± 0.02 | 34 ± 4 | 12 ± 3 |
|  | 25 | ─ | 11 ± 1.1 | 0.2 ± 0.01 | 3.8 ± 0.2 | 0.4 ± 0.2 |
|  | 5 | + | 2.5 ± 0.5 | 3.7 ± 0.03 | 74 ± 5 | 39 ± 6 |
|  | 25 | + | 9.1 ± 0.8 | 0.3 ± 0.01 | 5.8 ± 0.9 | 0.6 ± 0.1 |
| *P. aeruginosa* | 5 | ─ | 0.7 ± 0.1 | 1.5 ± 0.03 | 29 ± 1 | 40 ± 4 |
|  | 25 | ─ | 1.1 ± 0.2 | 1.0 ± 0.02 | 21 ± 2 | 18 ± 4 |
|  | 5 | + | 2.3 ± 0.4 | 3.9 ± 0.01 | 78 ± 3 | 34 ± 5 |
|  | 25 | + | 0.9 ± 0.2 | 2.3 ± 0.01 | 46 ± 1 | 52 ± 7 |
| *K. pneumoniae* | 5 | ─ | 0.9 ± 0.1 | 2.4 ± 0.01 | 48 ± 2 | 51 ± 6 |
|  | 25 | ─ | 2.1 ± 0.1 | 0.6 ± 0.01 | 12 ± 2 | 6 ± 3 |
|  | 5 | + | 4.6 ± 0.5 | 6.0 ± 0.03 | 120 ± 6 | 26 ± 3 |
|  | 25 | + | 1.6 ± 0.2 | 4.3 ± 0.02 | 86 ± 4 | 53 ± 8 |
|  |  |  |  |  |  |  |

**Table S1. Kinetic parameters of α-thrombin in the absence or presence of bacterial LPS.** Assays were performed at 37°C in 50 mM Tris-HCl buffer containing 0.15 M NaCl (pH 7.4), with or without 5 mM CaCl_2_ (Ca^2+^ presence indicated by “+”). Reactions were initiated by the addition of thrombin (0.5 nM) to the substrate (10 μM). Data represent means ± standard deviation (SD) from three independent experiments. K_M_, Michaelis constant; V_max_, maximum reaction velocity; k_cat_, turnover number; k_cat_/K_M_, catalytic efficiency.


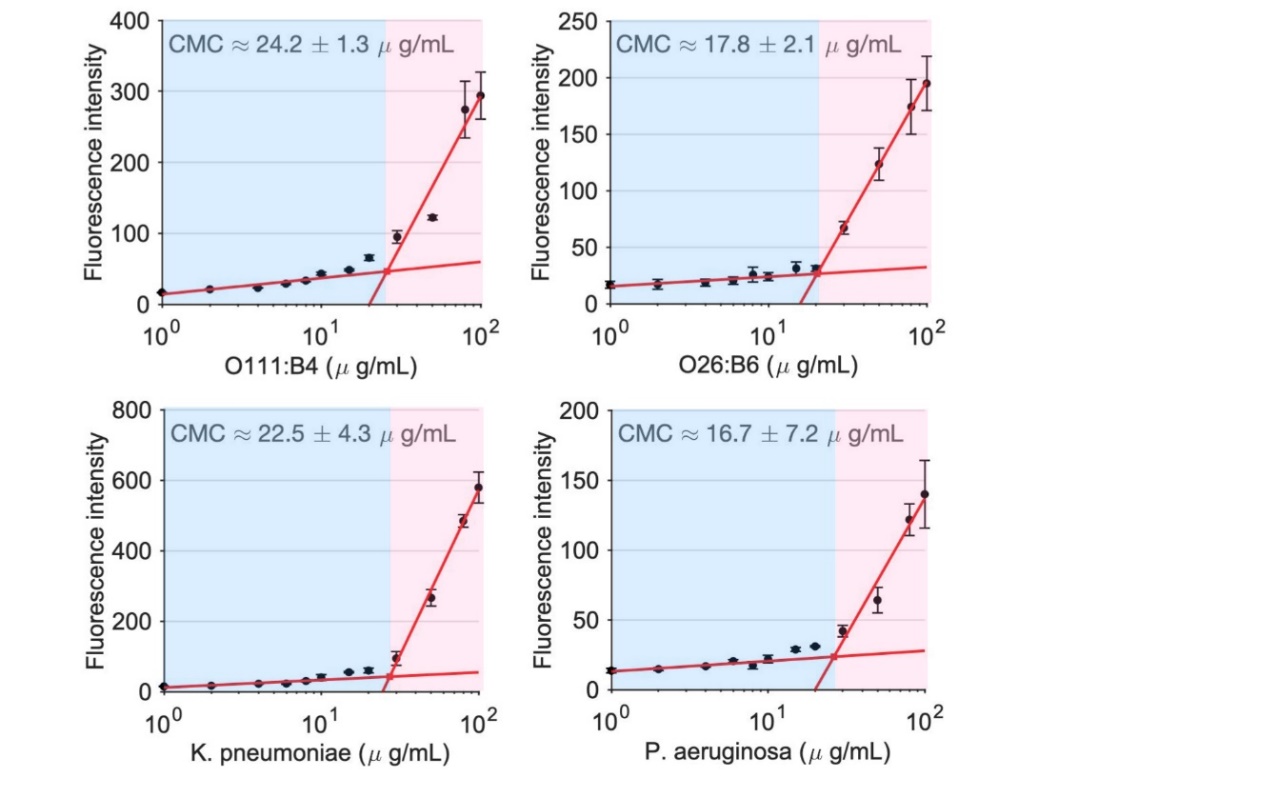


B

A

D

C

**Figure S1. Critical micelle concentration (CMC) of LPS chemotypes.** CMC values for (a) *E. coli* O111:B4, (b) *E. coli* O26:B6, (c) *P. aeruginosa*, and (d) *K. pneumoniae* were measured at 25 °C in 20 mM HEPES buffer containing 150 mM NaCl, pH 7.4. NPN fluorescence was monitored with excitation at 350 nm, and a sharp increase in intensity was observed near the CMC. The CMC was determined from the intersection of the two slope regions in the fluorescence curve. Shaded blue areas represent LPS in the monomeric/oligomeric state, whereas red areas correspond to micellar aggregates.


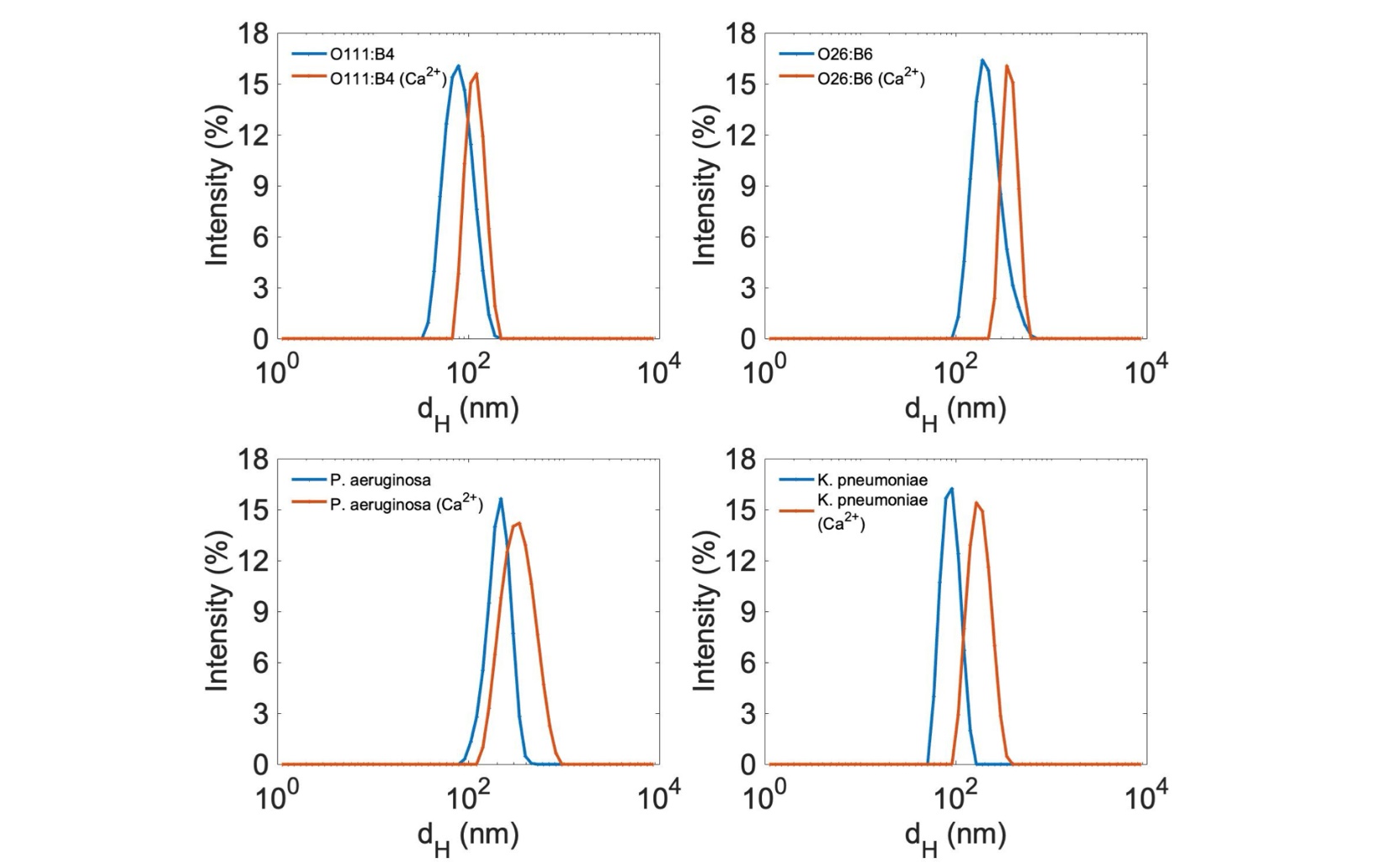


B

A

D

C

**Figure S2. Size distribution of LPS chemotype aggregates.** Hydrodynamic diameter (dh) profiles of LPS aggregates are shown for (A) *E. coli* O111:B4 (blue) and O111:B4 in the presence of Ca²⁺ (red), (B) *E. coli* O26:B6 (blue) and O26:B6 (Ca²⁺) (red), (C) *P. aeruginosa* (blue) and *P. aeruginosa* (Ca²⁺) (red), and (D) *K. pneumoniae* (blue) and *K. pneumoniae* (Ca²⁺) (red). Peak hydrodynamic diameters were 81.5 ± 5.0 nm and 129.1 ± 9.2 nm (O111:B4), 223.1 ± 14.1 nm and 342.3 ± 34.1 nm (O26:B6), 213.2 ± 31.0 nm and 339.4 ± 44.7 nm (*P. aeruginosa*), and 91.7 ± 11.1 nm and 163.2 ± 13.5 nm (*K. pneumoniae*), respectively. Measurements were performed in 20 mM HEPES buffer, pH 7.4.


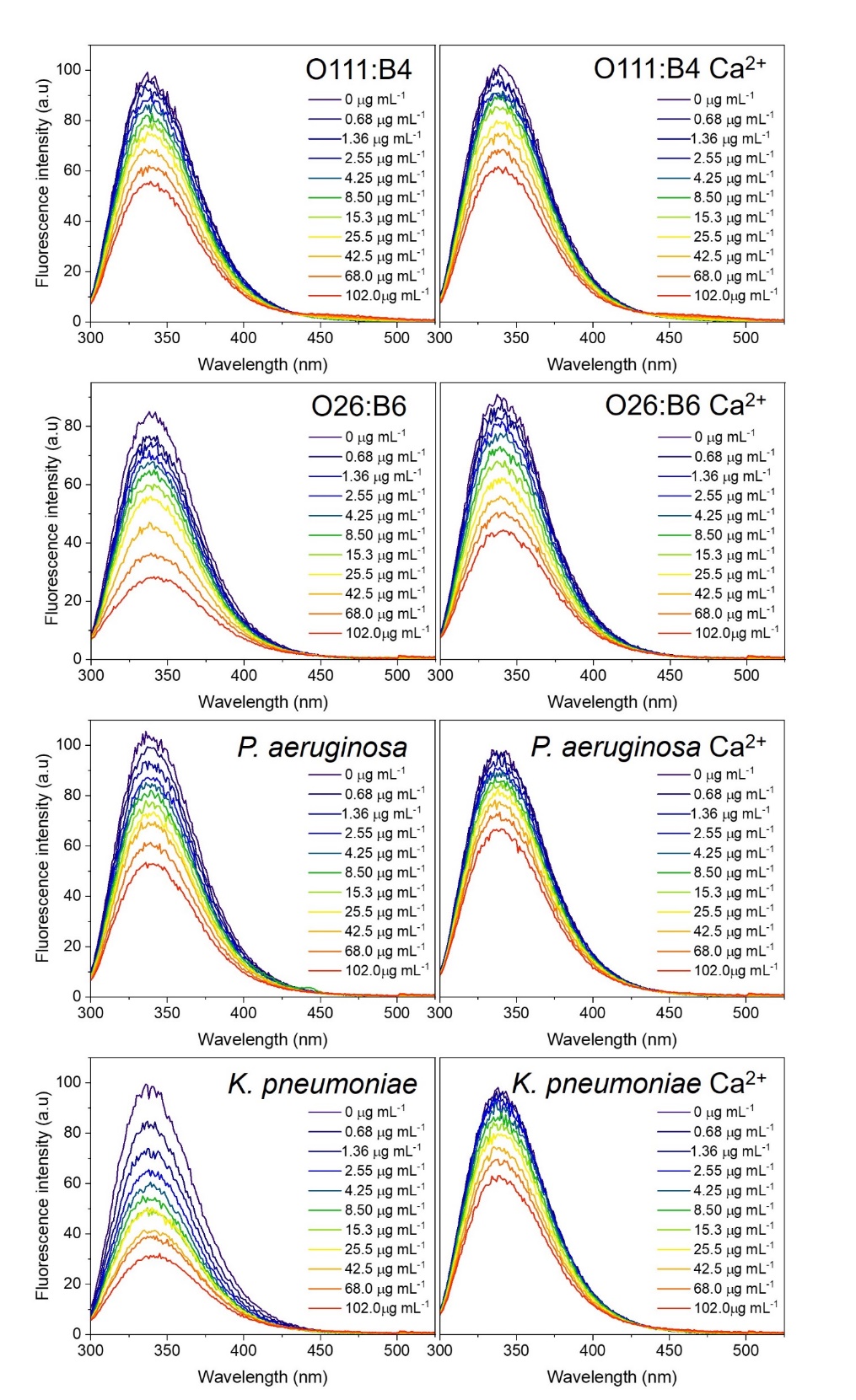


A

B

C

D

**Figure S3. Quenching of α-thrombin intrinsic fluorescence by LPS chemotypes.**The intrinsic fluorescence of α-thrombin (0.5 μM), excited at 280 nm, was measured in the presence of increasing concentrations of LPS from *E. coli* O111:B4 (A), *E. coli* O26:B6 (B), *P. aeruginosa* (C), and *K. pneumoniae* (D), with or without Ca²⁺. Progressive decreases in fluorescence intensity were observed, indicating interactions between thrombin and each LPS chemotype. Data represent mean ± SD from three independent experiments.

**
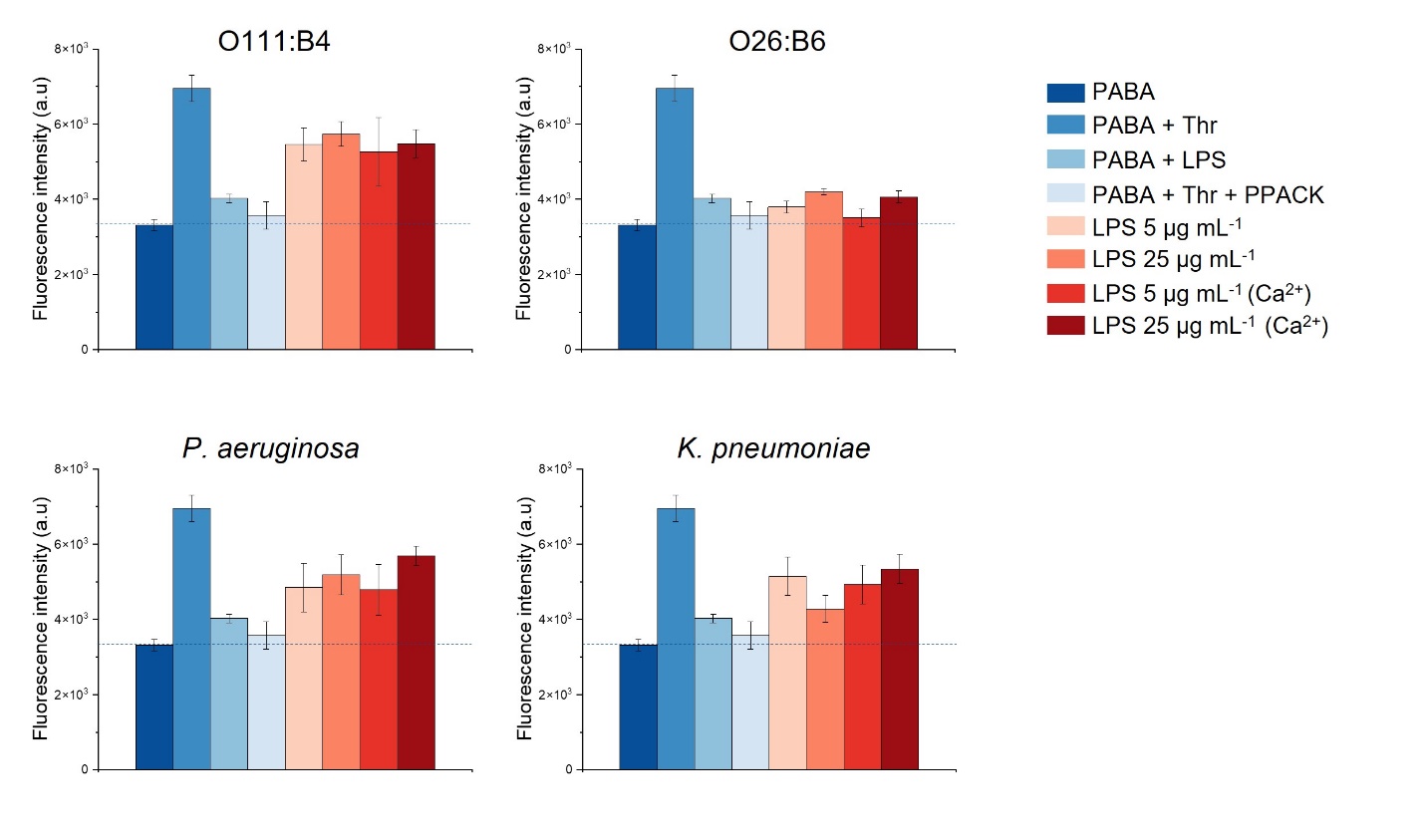
Figure S4. LPS effect on PABA binding to thrombin.** PABA fluorescence was measured in black 96-well plates using a Tecan plate reader under the following conditions: buffer alone; LPS alone (5 or 25 µg mL^-1^, prepared with or without Ca²⁺); thrombin (0.5 μM); thrombin preincubated with 1 μM PPACK; and thrombin with LPS. Free PABA exhibited low fluorescence, which was not altered by LPS alone. In contrast, addition of thrombin markedly enhanced PABA fluorescence, reflecting increased quantum yield upon active-site binding. The signal was reduced to baseline when thrombin was inhibited with PPACK, confirming specific occupation of the catalytic site. Different LPS chemotypes modulated PABA binding in distinct ways: *E. coli* O111:B4 and *P. aeruginosa* LPS maintained active-site accessibility with slightly reduced intensity, whereas *E. coli* O26:B6 and micellar *K. pneumoniae* LPS (25 µg mL^-1^) largely blocked PABA binding. Measurements were performed in HEPES buffer (150 mM NaCl), with excitation and emission wavelengths of 345 and 370 nm, respectively.


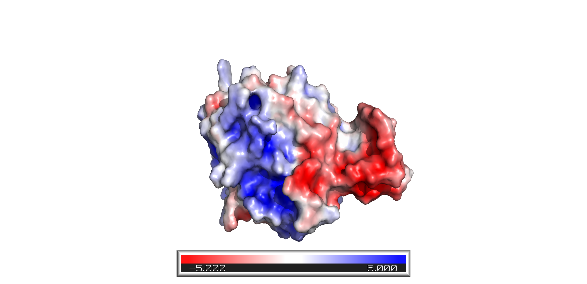

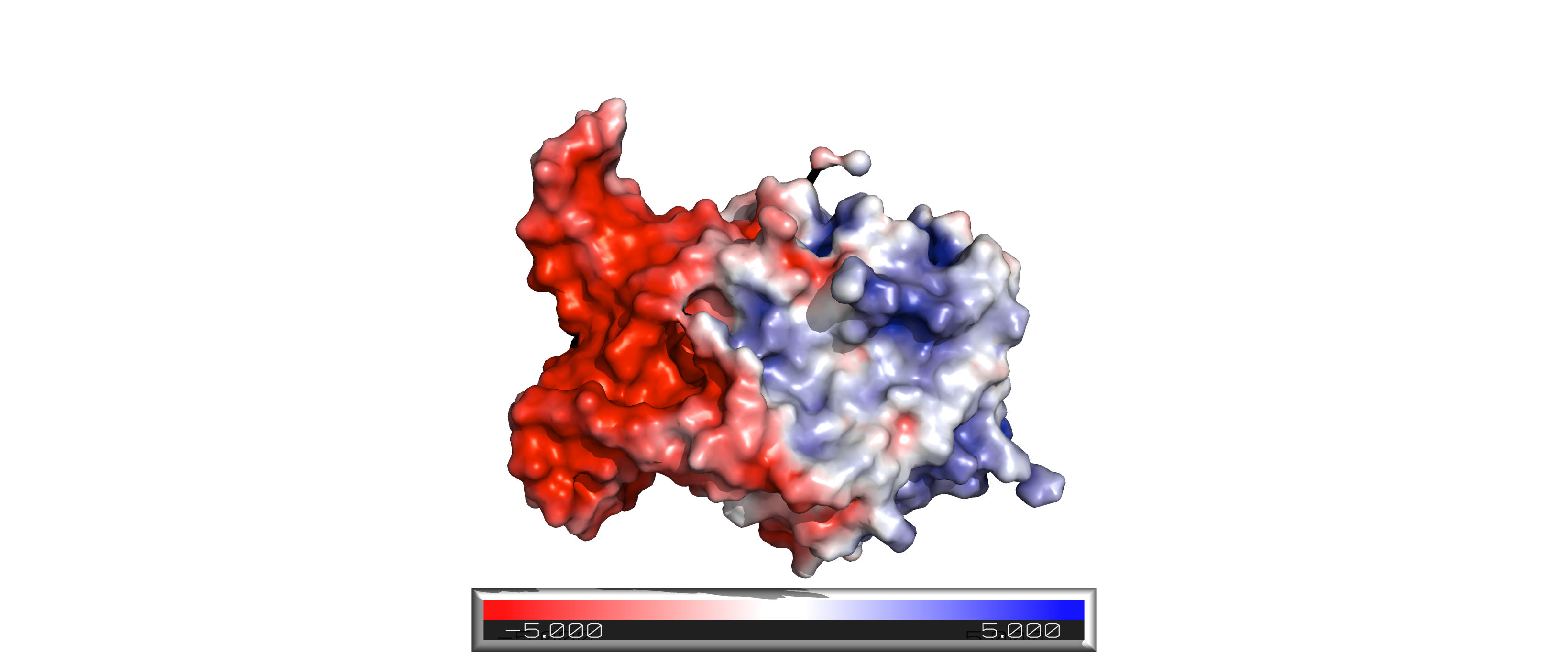

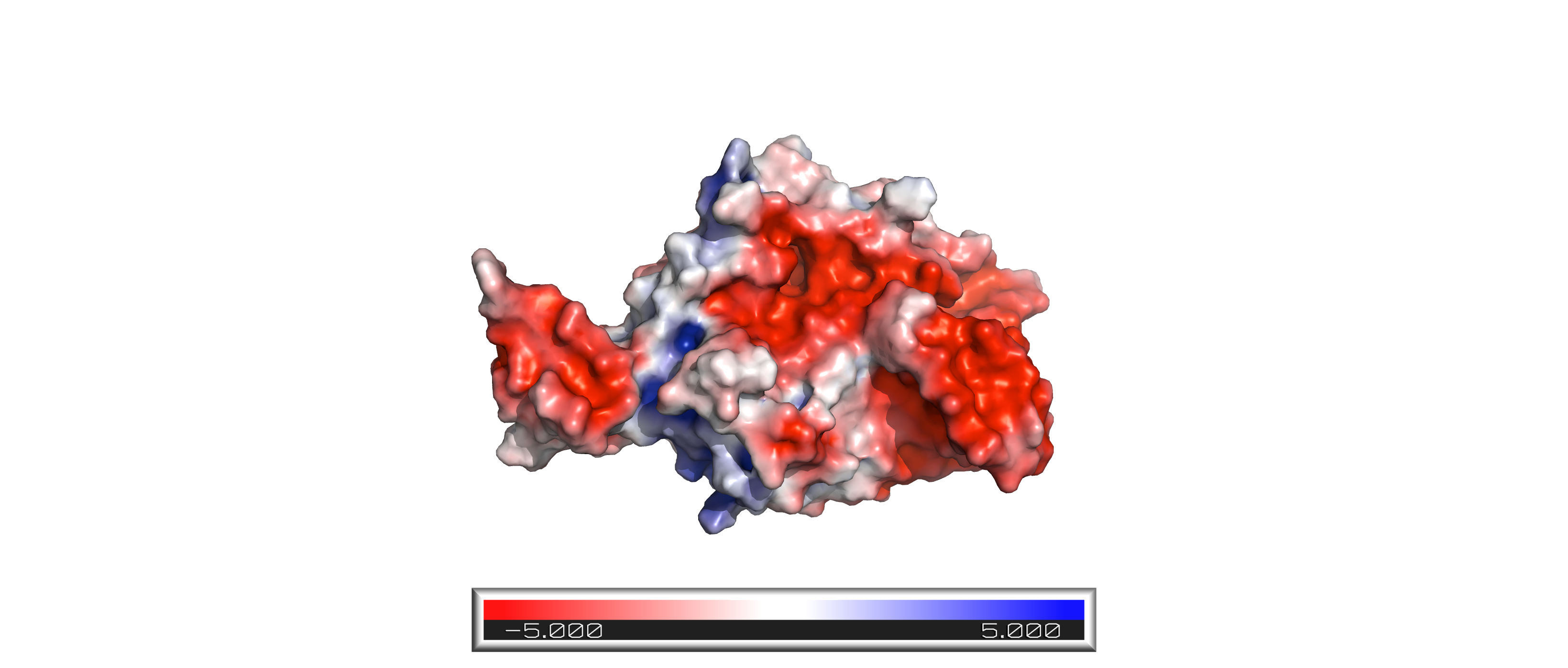


+ 5 kT/e

- 5 kT/e

HD1 + thrombin

HD1 + HD22 + thrombin

HD22 + thrombin

**Figure S5.** **Thrombin–aptamer electrostatic surface**. Molecular surface of human α-thrombin bound to HD1, HD22, and the combination of HD1+HD22 heterodimer (PDB: 6Z8X, 8TQS, 5EW1, respectively). Surfaces are colored by electrostatic potential (red: acidic; blue: basic; white: neutral). Both aptamers occupy highly electropositive regions, potentially blocking LPS binding via steric and electrostatic hindrance.


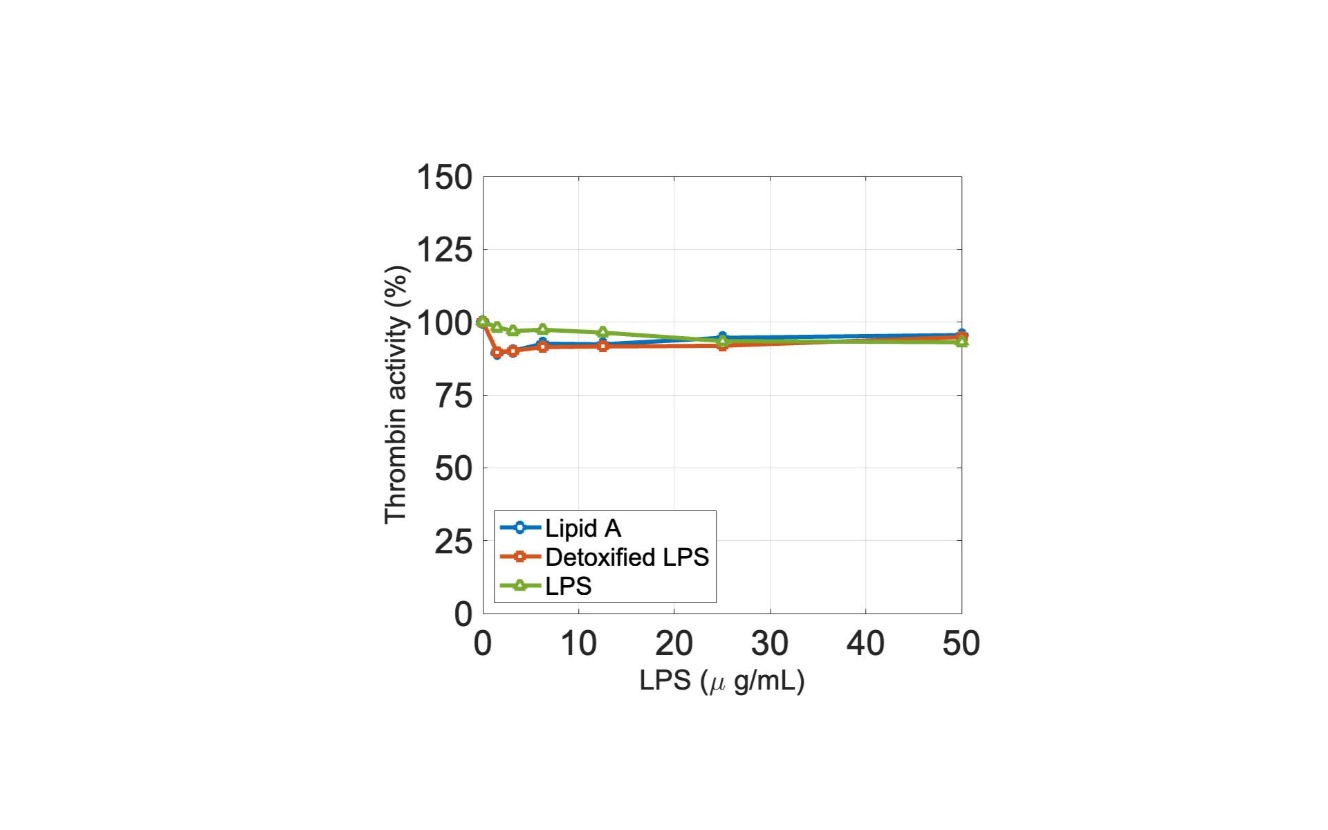


**Figure S6. Concentration-dependent inhibition of thrombin by D-LPS and lipid A.** Thrombin activity (5 nM) was assessed in the presence of increasing concentrations (0–50 μg/mL) of LPS O111:B4 (green), detoxified LPS (D-LPS, red), or lipid A (blue). Reactions contained 200 μM S-2238 in HEPES buffer supplemented with 150 mM NaCl. No inhibition of thrombin activity was detected under the conditions tested.


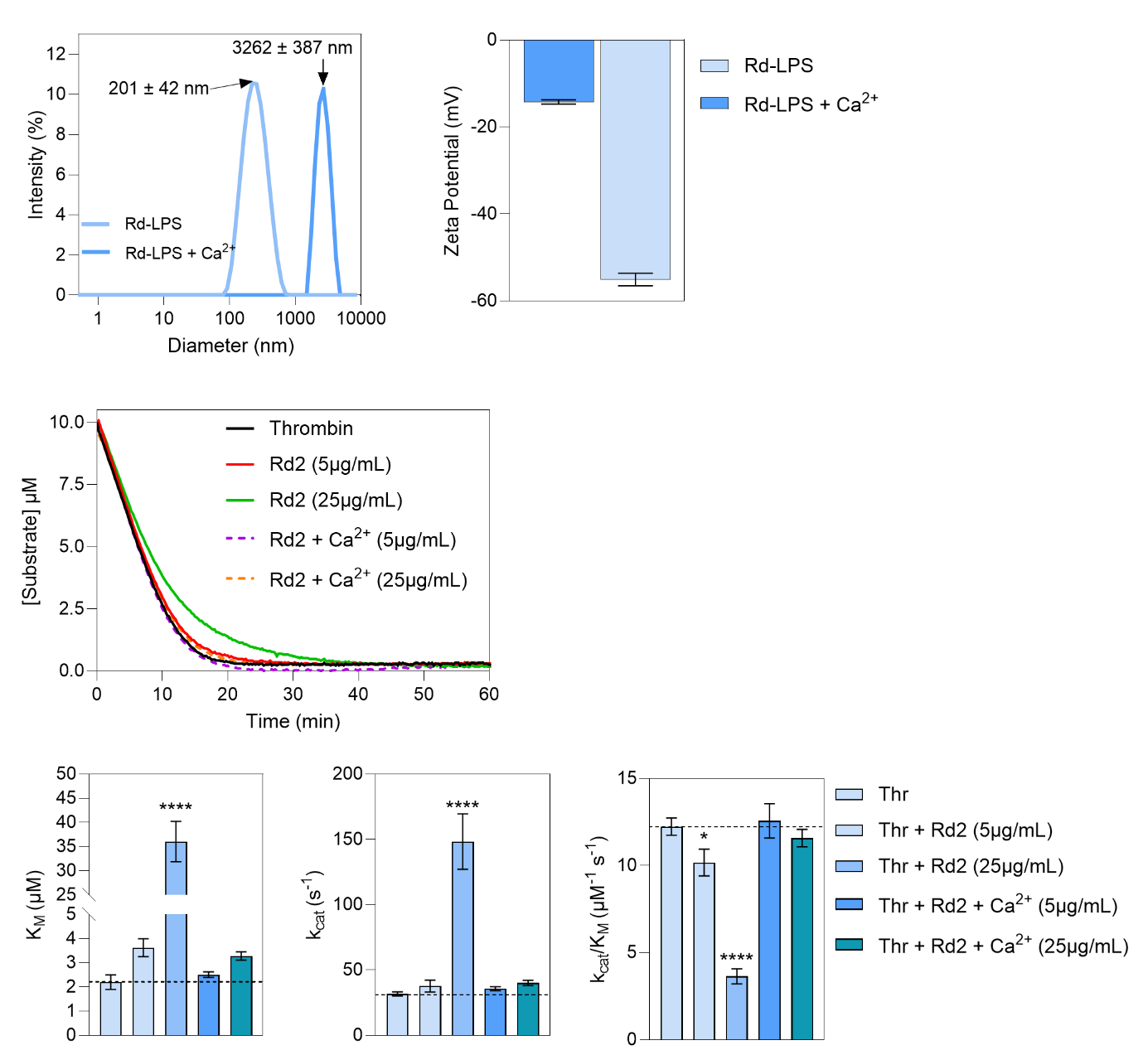


C

B

A

**Figure S7**. **Physicochemical characterization of rough LPS (Rd2) and its effects on thrombin progress-curve.** (a) Dynamic light scattering (DLS) size distributions of Rd2-LPS micelles in the absence or presence of Ca^2+^ (left; peak hydrodynamic diameters indicated) and corresponding ζ-potential measurements (right). (b) Representative thrombin progress curves showing time-dependent substrate depletion for thrombin alone or in the presence of Rd2-LPS (5 or 25 μg/mL), with or without Ca^2+^. (c) Apparent kinetic parameters (K_M_, k_cat_, and k_cat_/K_M_) obtained by fitting the progress-curve data to the explicit Schnell–Mendoza integrated Michaelis–Menten equation; the dashed line marks the Apparent kinetic parameters of thrombin alone. Error bars indicate variability among replicates, and significance versus thrombin control is denoted by ****p < 0.0001.

A

BB


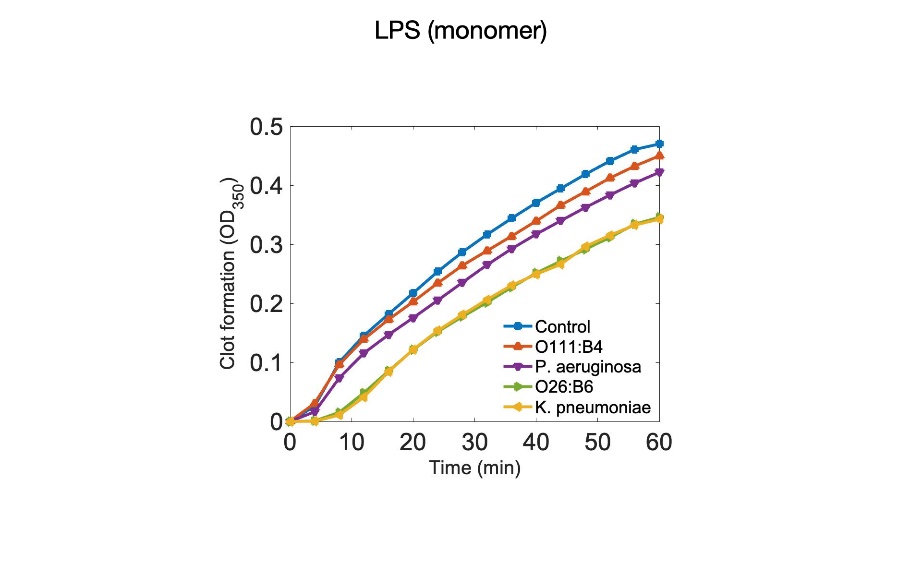

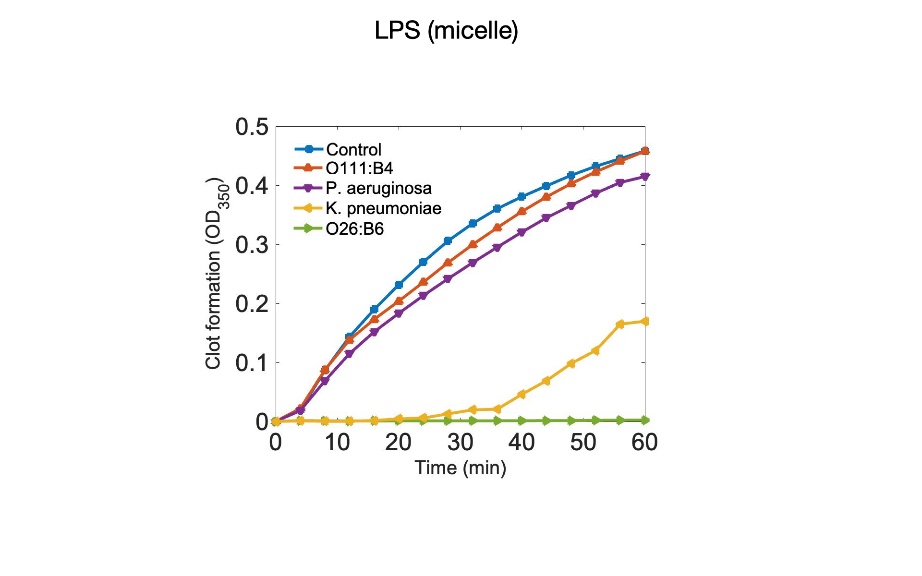

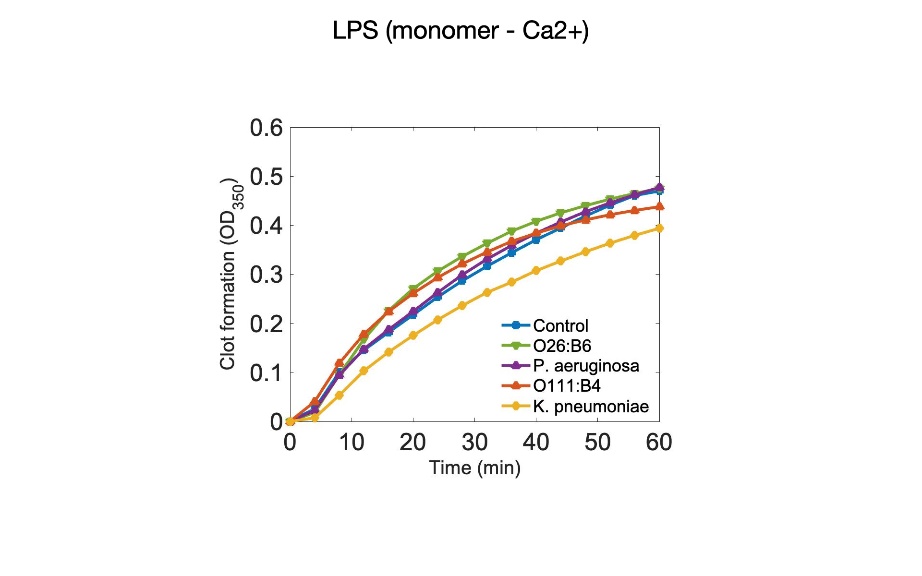

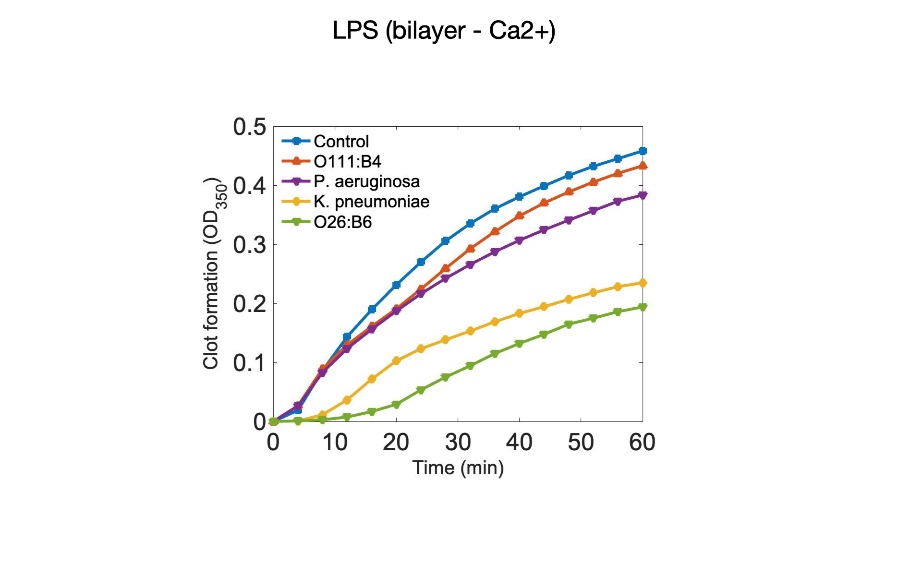


Monomer

Micelle

Bilayer-fragment

Bilayer

D

C

**Figure S8**. **Time course of fibrin clot formation by turbidity measurements.** Turbidity assays illustrating the effect of different LPS chemotypes on fibrin clot formation in various structural states. Thrombin (5 nM) was pre-incubated with different LPS chemotypes (indicated in the legends) at 5 or 25 ug/mL in HEPES buffer, either alone or supplemented with Ca2+ for 15 minutes at 37C. Clotting was initiated by adding fibrinogen (4 uM) and fibrin formation was monitored over time by measuring turbidity at 350 nm (OD350). Control wells lacking LPS were included. All concentrations represent final values after mixing. The different panels illustrate the effect of LPS under conditions that favor distinct LPS structural aggregates: bilayer (LPS ), monomer – Ca2+ (LPS, low ca2+), micelle (LPS, high detergent or low ), and monomer (LPS, low or low concentration). Clot formation is expressed as OD350.


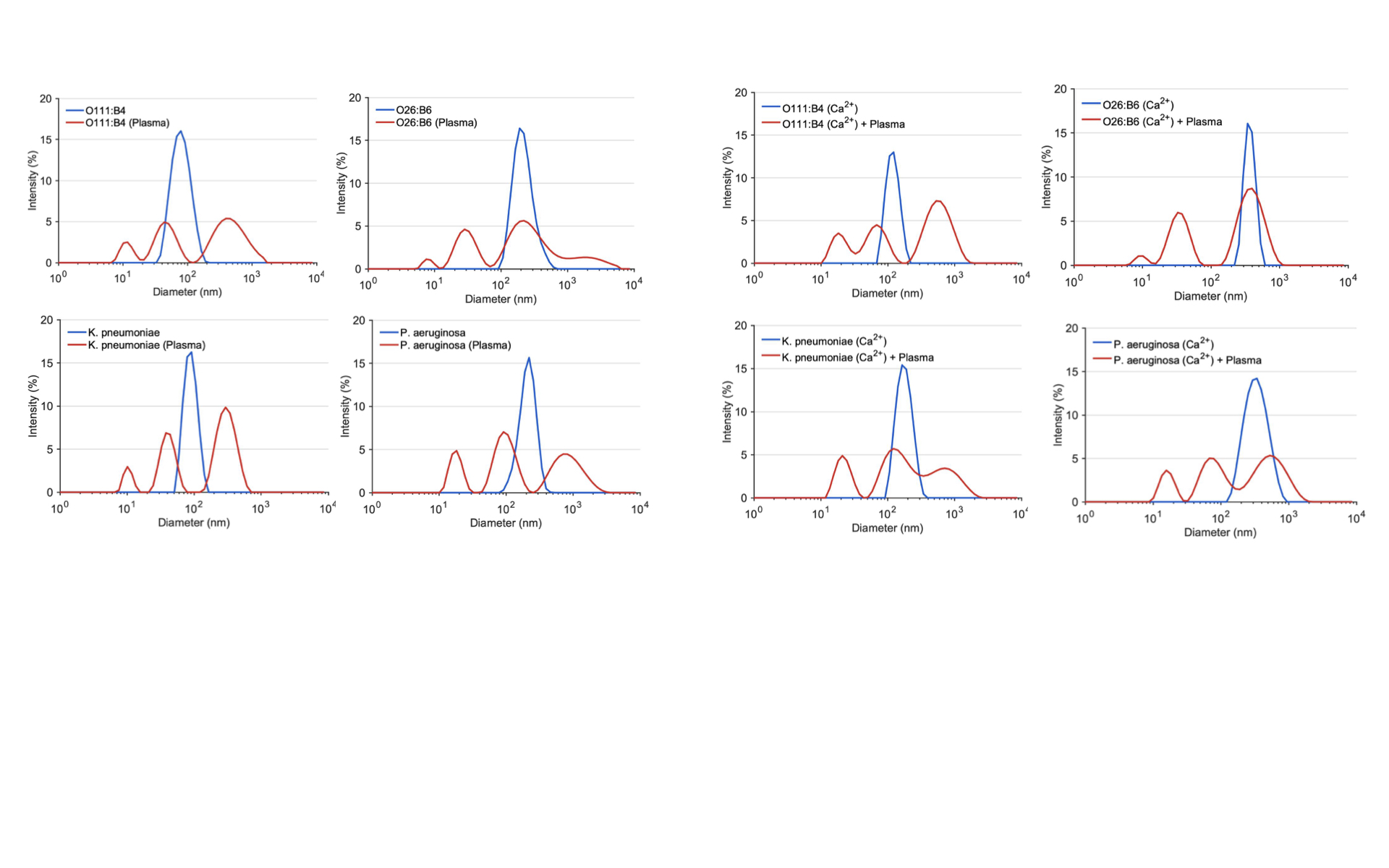


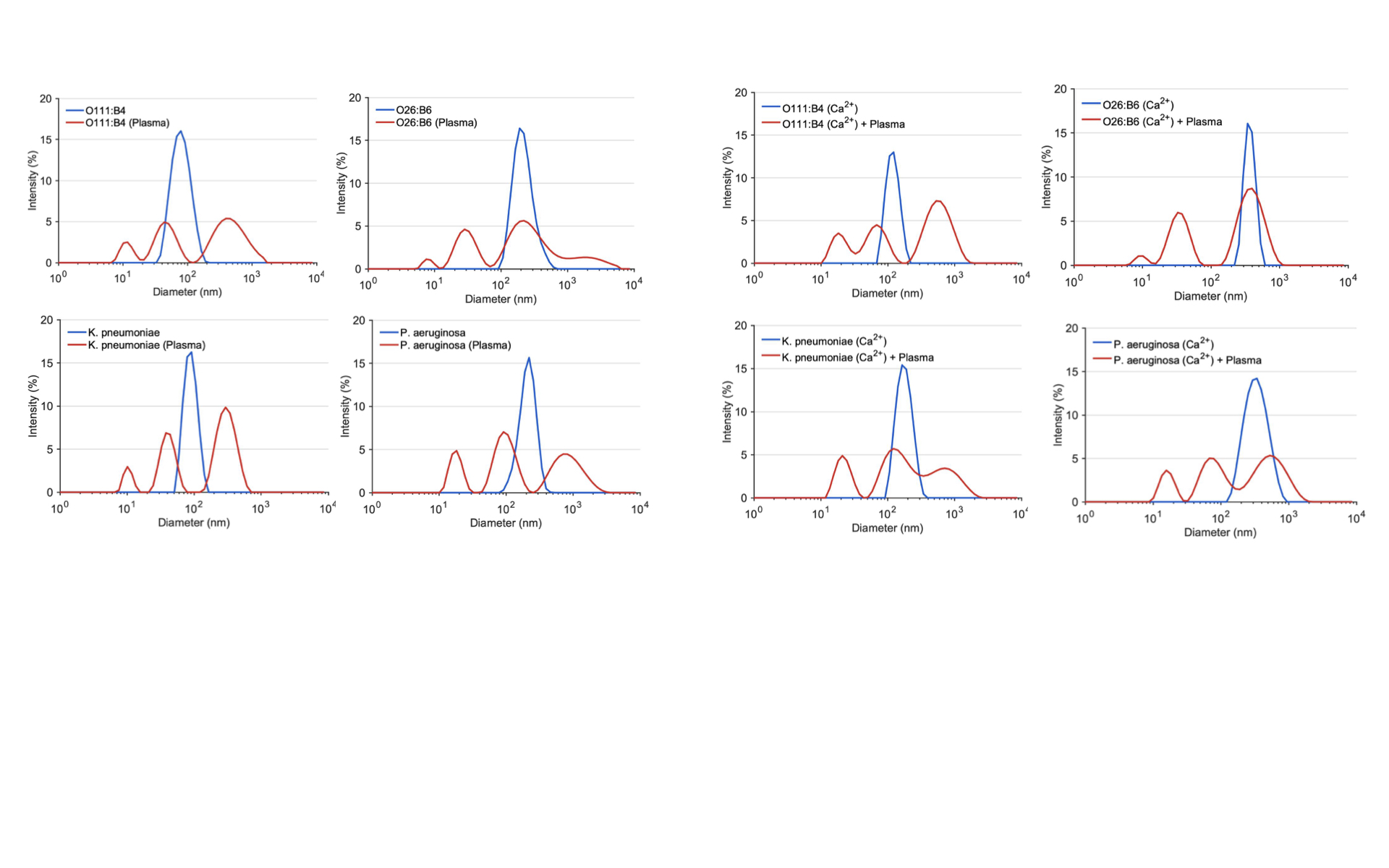


**Figure S9.** **Size distribution of LPS in human plasma.** LPS aggregates prepared in the presence or absence of Ca^2+^ were analyzed in buffer (blue) and human plasma (red). The reduced aggregate size in plasma likely reflects disaggregation by plasma proteins (e.g., albumin) and the formation of a biomolecular corona.
